# Supplementary material for: The HTAP_v3 emission mosaic: merging regional and global monthly emissions (2000–2018) to support air quality modelling and policies
Source: Earth Syst Sci Data. Author manuscript; Available in PMC 2025 Jan 2. (PMC11694899; doi:10.5194/essd-15-2667-2023)
Supplement: Supplement1 [file NIHMS2032476-supplement-Supplement1.pdf]

Supplement of Earth Syst. Sci. Data, 15, 2667–2694, 2023
https://doi.org/10.5194/essd-15-2667-2023-supplement
© Author(s) 2023. CC BY 4.0 License.

Supplement of

The HTAP_v3 emission mosaic: merging regional and global monthly emissions (2000–2018) to support air quality modelling and policies

Monica Crippa et al.

Correspondence to: Marilena Muntean (marilena.muntean@ec.europa.eu) and Monica Crippa (monica.crippa@ext.ec.europa.eu)

The copyright of individual parts of the supplement might differ from the article licence.

S1 - Matching tables between inventory providers and HTAP_v3 sectors and regions

Table S1 – Sector matching table between inventory providers and HTAP_v3 sectors

	REASv3.2.1	CAPSS-KU	JAPAN	US EPA	ECCC	CAMS-REGv6.1
**HTAP_1: International Shipping**	-	-	-	-	-	-
**HTAP_2.1: Domestic Aviation**		SCC Level3 domestic airport traffic	1.A.3.a.i.(i) Civil aviation (domestic, landing/take-off (LTO))	1A3aii_Domestic-aviation	Landing & Takeoff 1A3ai(i) and 1A3aii(i)	H_Aviation, O_AviCruise
**HTAP_2.2: International Aviation**	-	-	-	-	-	-
**HTAP_3: Energy**	POWER_PLANTS_POINT POWER_PLANTS_NON-POINT	SCC Level1 Energy Industry (Power Generation)	1.A.1.a Public electricity and heat production	1A1a_Public-Electricity 1A1a_Industrial-Electricity	1A1a_Public-Electricity 1A1a_Industrial-Electricity	A_PublicPower
**HTAP_4.1: Industry**	INDUSTRY	SCC Level1 Combustion in manufacturing industry SCC Level1 Industrial processes	1.A.2 All components 2.A Mineral products 2.B Chemical industry 2.C Metal production 2.H Other industry production	1A2 All components 2 All components (excluding 2D) 6A_Other-commertial	1A2 All components, excluding Fugitive 2 All components (excluding 2D & excluding Fugitive)	B_Industry

**HTAP_4.2: Fugitive**	EXTRACTION	SCC Level1 Energy storage and distribution<br>SCC Level2 charcoal manufacturing	1.A.1.c Manufacture of solid fuels and other energy industries<br>1.A.1.b Petroleum refining<br>1.B Fugitive emissions from fuels	1B2 All components<br>1A1b_Pet-refining<br>1A1c_Coke-ovens<br>1A1g_Other-energy-transf	Fugitive components of 1A2<br>1B2 All components<br>2A5	D_Fugitive
**HTAP_4.3: Solvents**	SOLVENT	SCC Level1<br>Solvent use	2.D Other solvent and product use	2D All components	Solvent portions of 1A2, 1B2, 2D and 2L	E_Solvents
**HTAP_5.1: Road Transport**	ROAD_TRANSPORT	on-road mobile	1.A.3.b.i, ii, iii, iv, v Road transport	1A3bii_Road (combustion)	1A3bi – iv	F_RoadTransport (excluding resuspension)
**HTAP_5.2: Brake and Tyre wear**	-	SCC Level2 paved road<br>SCC Level2 unpaved road<br>SCC Level2 tire wear	1.A.3.b.vi, vii Road transport	1A3b_Road-noncomb	1A3bv – vii<br>6A Road Dust	F_RoadTransport (resuspension only)
**HTAP_5.3: Domestic shipping**	-	SCC Level2 Inland waterways<br>SCC Level3 national sea traffic within EMEP area	-	1A3dii_Domestic-navigation (shipping)<br>1A5_Recreational-Equipment-Marine	1A3dii	G_Shipping
**HTAP_5.4: Other ground transport**	OTHER_TRANSPORT	SCC Level2 Railways	1.A.3.c Railways	1A3c_Rail<br>1A3eii_Other-unspecified-transp<br>1A5_Recreational-Equipment-Land	1A2gvii Off road<br>1A3c<br>1A3ei<br>1A4bii	I_Offroad

**HTAP_6: Residential**	DOMESTIC	SCC Level1 Non-industrial combustion plants<br>SCC Level2 Agriculture<br>SCC Level2 construction machinery<br>SCC Level2 fireplace	1.A.4 All components	1A4 All components	1A4 All components<br>2D3e Commercial residential<br>2H2 Meat grilling<br>6A cigarette smoking,<br>Structural fires	C_OtherStationary Comb
**HTAP_7: Waste**	-	SCC Level1 Waste treatment and disposal<br>SCC Level2 waste disposal	5. Waste	5 All components	5 All components	J_Waste
**HTAP_8.1: Agricultural waste burning**	-	SCC Level2 agricultural residue burning	3.F Field burning of agricultural residues	3F_Ag-res-on-field	6A Prescribed Burning (forest)	L_AgriOther (agricultural waste burning only)
**HTAP_8.2: Agriculture_livestock**	MANURE_MANAGEMENT	SCC Level2 Manure management<br>SCC Level2 Stockbreeding activity	3.B Manure management	3B All components	3B All components	K_AgriLivestock
**HTAP_8.3: Agriculture_crops**	FERTILIZER	SCC Level2 Cultures with fertilizers<br>(except animal manure)<br>SCC Level2 Agriculture activity	3.D Crop production and agricultural soils	3Da1_Inorganic-N-fertilizers<br>3Df_Use-of-pesticides	3D All components	L_AgriOther (excluding agricultural waste burning)

Table S2- Matching sectors between MEIC and HTAP_v3

While the HTAP_v3 mosaic includes 16 separate sectors, the public MEIC inventory is only distributed with 4 aggregated sectors. HTAP_v3 aims to be a “complete” inventory, covering all known anthropogenic emission sources, while the scope of the MEIC inventory is more limited. Some of the HTAP_v3 sectors have no corresponding counterpart in MEIC, while others are present in MEIC as part of aggregated sectors. To compare the HTAP_v3 emissions with MEIC emissions, the correspondence between sectors must be established.

HTAP_v3 sector number	HTAP_v3 sector name	MEIC sector
1	International Shipping	Not included in MEIC
2.1	Domestic Aviation	Not included in MEIC
2.2	International Aviation	Not included in MEIC
3	Energy	Power
4.1	Industry	Industry
4.2	Fugitive	Industry
4.3	Solvents	Industry
5.1	Road Transport	Transportation
5.2	Brake and Tyre wear	Transportation
5.3	Domestic shipping	Transportation
5.4	Other ground transport	Transportation
6	Residential	Residential
7	Waste	Residential
8.1	Agricultural waste burning	Not included in MEIC
8.2	Agriculture livestock	Agriculture
8.3	Agriculture crops	Agriculture

Table S3 – Country mapping to inventory providers and regional belonging to IPCC AR6 regions.

Data provider	Country code ISO_A3	Country name	IPCC AR6 regional grouping
CAMS-REG-v5.1	ALB	Albania	Europe
CAMS-REG-v5.1	AUT	Austria	Europe
CAMS-REG-v5.1	BEL	Belgium	Europe
CAMS-REG-v5.1	BGR	Bulgaria	Europe
CAMS-REG-v5.1	BIH	Bosnia and Herzegovina	Europe
CAMS-REG-v5.1	BLR	Belarus	Eurasia
CAMS-REG-v5.1	CHE	Switzerland	Europe
CAMS-REG-v5.1	CYP	Cyprus	Europe
CAMS-REG-v5.1	CZE	Czech Republic	Europe
CAMS-REG-v5.1	DEU	Germany	Europe
CAMS-REG-v5.1	DNK	Denmark	Europe
CAMS-REG-v5.1	ESP	Spain	Europe
CAMS-REG-v5.1	EST	Estonia	Europe
CAMS-REG-v5.1	FIN	Finland	Europe
CAMS-REG-v5.1	FRA	France	Europe
CAMS-REG-v5.1	GBR	United Kingdom	Europe
CAMS-REG-v5.1	GRC	Greece	Europe
CAMS-REG-v5.1	HRV	Croatia	Europe
CAMS-REG-v5.1	HUN	Hungary	Europe
CAMS-REG-v5.1	IRL	Ireland	Europe
CAMS-REG-v5.1	ISL	Iceland	Europe
CAMS-REG-v5.1	ITA	Italy	Europe

CAMS-REG-v5.1	LTU	Lithuania	Europe
CAMS-REG-v5.1	LUX	Luxembourg	Europe
CAMS-REG-v5.1	LVA	Latvia	South-East Asia and developing Pacific
CAMS-REG-v5.1	MDA	Moldova, Republic of	Eurasia
CAMS-REG-v5.1	MKD	Macedonia, the former Yugoslav Republic of	Eurasia
CAMS-REG-v5.1	MLT	Malta	Europe
CAMS-REG-v5.1	MNE	Montenegro	Europe
CAMS-REG-v5.1	NLD	Netherlands	Europe
CAMS-REG-v5.1	NOR	Norway	Europe
CAMS-REG-v5.1	POL	Poland	Europe
CAMS-REG-v5.1	PRT	Portugal	Europe
CAMS-REG-v5.1	ROU	Romania	Europe
CAMS-REG-v5.1	SRB	Serbia	Europe
CAMS-REG-v5.1	SVK	Slovakia	Europe
CAMS-REG-v5.1	SVN	Slovenia	Europe
CAMS-REG-v5.1	SWE	Sweden	Europe
CAMS-REG-v5.1	TUR	Turkey	Europe
CAMS-REG-v5.1	UKR	Ukraine	Europe
CAMS-REG-v5.1	XKX	Kosovo	Europe
CAPSS-KU	KOR	Korea, Republic of	Eastern Asia
ECCC	CAN	Canada	North America
EDGARv6.1	ABW	Aruba	Latin America and Caribbean
EDGARv6.1	AGO	Angola	Africa
EDGARv6.1	AIA	Anguilla	Latin America and Caribbean
EDGARv6.1	AIR	Int. Aviation	Int. Aviation
EDGARv6.1	ANT	Netherlands Antilles	Latin America and Caribbean
EDGARv6.1	ARE	United Arab Emirates	Middle East
EDGARv6.1	ARG	Argentina	Latin America and Caribbean
EDGARv6.1	ARM	Armenia	Eurasia
EDGARv6.1	ASM	American Samoa	South-East Asia and developing Pacific

EDGARv6.1	ATG	Antigua and Barbuda	Latin America and Caribbean
EDGARv6.1	AUS	Australia	Asia-Pacific Developed
EDGARv6.1	AZE	Azerbaijan	Eurasia
EDGARv6.1	BDI	Burundi	Africa
EDGARv6.1	BEN	Benin	Africa
EDGARv6.1	BFA	Burkina Faso	Africa
EDGARv6.1	BHR	Bahrain	Middle East
EDGARv6.1	BHS	Bahamas	Latin America and Caribbean
EDGARv6.1	BLZ	Belize	Latin America and Caribbean
EDGARv6.1	BMU	Bermuda	Latin America and Caribbean
EDGARv6.1	BOL	Bolivia	Latin America and Caribbean
EDGARv6.1	BRA	Brazil	Latin America and Caribbean
EDGARv6.1	BRB	Barbados	Latin America and Caribbean
EDGARv6.1	BWA	Botswana	Africa
EDGARv6.1	CAF	Central African Republic	Africa
EDGARv6.1	CHL	Chile	Latin America and Caribbean
EDGARv6.1	CIV	Cote d'Ivoire	Africa
EDGARv6.1	CMR	Cameroon	Africa
EDGARv6.1	COD	Congo_the Democratic Republic of the	Africa
EDGARv6.1	COG	Congo	Africa
EDGARv6.1	COK	Cook Islands	South-East Asia and developing Pacific
EDGARv6.1	COL	Colombia	Latin America and Caribbean
EDGARv6.1	COM	Comoros	Africa
EDGARv6.1	CPV	Cape Verde	Africa
EDGARv6.1	CRI	Costa Rica	Latin America and Caribbean
EDGARv6.1	CUB	Cuba	Latin America and Caribbean
EDGARv6.1	CYM	Cayman Islands	Latin America and Caribbean
EDGARv6.1	DJI	Djibouti	Africa
EDGARv6.1	DMA	Dominica	Latin America and Caribbean
EDGARv6.1	DOM	Dominican Republic	Latin America and Caribbean
EDGARv6.1	DZA	Algeria	Africa
EDGARv6.1	ECU	Ecuador	Latin America and Caribbean
EDGARv6.1	EGY	Egypt	Africa
EDGARv6.1	ERI	Eritrea	Africa
EDGARv6.1	ESH	Western Sahara	Africa
EDGARv6.1	ETH	Ethiopia	Africa
EDGARv6.1	FJI	Fiji	South-East Asia and developing Pacific
EDGARv6.1	FLK	Falkland Islands (Malvinas)	Latin America and Caribbean
EDGARv6.1	FRO	Faroe Islands	Europe
EDGARv6.1	FSM	Micronesia, Federated States of	South-East Asia and developing Pacific
EDGARv6.1	GAB	Gabon	Africa
EDGARv6.1	GEO	Georgia	Eurasia
EDGARv6.1	GHA	Ghana	Africa

EDGARv6.1	GIB	Gibraltar	Europe
EDGARv6.1	GIN	Guinea	Africa
EDGARv6.1	GLP	Guadeloupe	Latin America and Caribbean
EDGARv6.1	GMB	Gambia	Africa
EDGARv6.1	GNB	Guinea-Bissau	Africa
EDGARv6.1	GNQ	Equatorial Guinea	Africa
EDGARv6.1	GRD	Grenada	Latin America and Caribbean
EDGARv6.1	GRL	Greenland	Europe
EDGARv6.1	GTM	Guatemala	Latin America and Caribbean
EDGARv6.1	GUF	French Guiana	Latin America and Caribbean
EDGARv6.1	GUM	Guam	South-East Asia and developing Pacific
EDGARv6.1	GUY	Guyana	Latin America and Caribbean
EDGARv6.1	HKG	Hong Kong	Eastern Asia
EDGARv6.1	HND	Honduras	Latin America and Caribbean
EDGARv6.1	HTI	Haiti	Latin America and Caribbean
EDGARv6.1	IRN	Iran, Islamic Republic of	Middle East
EDGARv6.1	IRQ	Iraq	Middle East
EDGARv6.1	ISR	Israel	Middle East
EDGARv6.1	JAM	Jamaica	Latin America and Caribbean
EDGARv6.1	JOR	Jordan	Middle East
EDGARv6.1	KAZ	Kazakhstan	Eurasia
EDGARv6.1	KEN	Kenya	Africa
EDGARv6.1	KGZ	Kyrgyzstan	Eurasia
EDGARv6.1	KIR	Kiribati	South-East Asia and developing Pacific
EDGARv6.1	KNA	Saint Kitts and Nevis	Latin America and Caribbean
EDGARv6.1	KWT	Kuwait	Middle East
EDGARv6.1	LBN	Lebanon	Middle East
EDGARv6.1	LBR	Liberia	Africa
EDGARv6.1	LBY	Libyan Arab Jamahiriya	Africa
EDGARv6.1	LCA	Saint Lucia	Latin America and Caribbean
EDGARv6.1	LSO	Lesotho	Africa
EDGARv6.1	MAC	Macao	Eastern Asia
EDGARv6.1	MAR	Morocco	Africa
EDGARv6.1	MDG	Madagascar	Africa
EDGARv6.1	MEX	Mexico	Latin America and Caribbean
EDGARv6.1	MLI	Mali	Africa
EDGARv6.1	MOZ	Mozambique	Africa
EDGARv6.1	MRT	Mauritania	Africa
EDGARv6.1	MSR	Montserrat	Latin America and Caribbean
EDGARv6.1	MTQ	Martinique	Latin America and Caribbean
EDGARv6.1	MUS	Mauritius	Africa
EDGARv6.1	MWI	Malawi	Africa
EDGARv6.1	MYT	Mayotte	Africa
EDGARv6.1	NAM	Namibia	Africa

EDGARv6.1	NCL	New Caledonia	South-East Asia and developing Pacific
EDGARv6.1	NER	Niger	Africa
EDGARv6.1	NGA	Nigeria	Africa
EDGARv6.1	NIC	Nicaragua	Latin America and Caribbean
EDGARv6.1	NIU	Niue	South-East Asia and developing Pacific
EDGARv6.1	NZL	New Zealand	Asia-Pacific Developed
EDGARv6.1	OMN	Oman	Middle East
EDGARv6.1	PAN	Panama	Latin America and Caribbean
EDGARv6.1	PER	Peru	Latin America and Caribbean
EDGARv6.1	PLW	Palau	South-East Asia and developing Pacific
EDGARv6.1	PNG	Papua New Guinea	South-East Asia and developing Pacific
EDGARv6.1	PRY	Paraguay	Latin America and Caribbean
EDGARv6.1	PYF	French Polynesia	South-East Asia and developing Pacific
EDGARv6.1	QAT	Qatar	Middle East
EDGARv6.1	REU	Reunion	Africa
EDGARv6.1	RUS	Russian Federation	Eurasia
EDGARv6.1	RWA	Rwanda	Africa
EDGARv6.1	SAU	Saudi Arabia	Middle East
EDGARv6.1	SDN	Sudan	Africa
EDGARv6.1	SEA	Int. Shipping	Int. Shipping
EDGARv6.1	SEN	Senegal	Africa
EDGARv6.1	SHN	Saint Helena	Africa
EDGARv6.1	SLB	Solomon Islands	South-East Asia and developing Pacific
EDGARv6.1	SLE	Sierra Leone	Africa
EDGARv6.1	SLV	El Salvador	Latin America and Caribbean
EDGARv6.1	SOM	Somalia	Africa
EDGARv6.1	SPM	Saint Pierre and Miquelon	North America
EDGARv6.1	STP	Sao Tome and Principe	Africa
EDGARv6.1	SUR	Suriname	Latin America and Caribbean
EDGARv6.1	SWZ	Swaziland	Africa
EDGARv6.1	SYC	Seychelles	Africa
EDGARv6.1	SYR	Syrian Arab Republic	Middle East
EDGARv6.1	TCA	Turks and Caicos Islands	Latin America and Caribbean
EDGARv6.1	TCD	Chad	Africa
EDGARv6.1	TGO	Togo	Africa
EDGARv6.1	TJK	Tajikistan	Eurasia
EDGARv6.1	TKL	Tokelau	South-East Asia and developing Pacific
EDGARv6.1	TKM	Turkmenistan	Eurasia
EDGARv6.1	TLS	Timor-Leste	South-East Asia and developing Pacific

EDGARv6.1	TON	Tonga	South-East Asia and developing Pacific
EDGARv6.1	TTO	Trinidad and Tobago	Latin America and Caribbean
EDGARv6.1	TUN	Tunisia	Africa
EDGARv6.1	TZA	Tanzania_United Republic of	Africa
EDGARv6.1	UGA	Uganda	Africa
EDGARv6.1	URY	Uruguay	Latin America and Caribbean
EDGARv6.1	UZB	Uzbekistan	Eurasia
EDGARv6.1	VCT	Saint Vincent and the Grenadines	Latin America and Caribbean
EDGARv6.1	VEN	Venezuela	Latin America and Caribbean
EDGARv6.1	VGB	Virgin Islands_British	Latin America and Caribbean
EDGARv6.1	VUT	Vanuatu	South-East Asia and developing Pacific
EDGARv6.1	WLF	Wallis and Futuna	South-East Asia and developing Pacific
EDGARv6.1	WSM	Samoa	South-East Asia and developing Pacific
EDGARv6.1	YEM	Yemen	Middle East
EDGARv6.1	ZAF	South Africa	Africa
EDGARv6.1	ZMB	Zambia	Africa
EDGARv6.1	ZWE	Zimbabwe	Africa
JAPAN	JPN	Japan	Asia-Pacific Developed
REAS	AFG	Afghanistan	Southern Asia
REAS	BGD	Bangladesh	Southern Asia
REAS	BRN	Brunei Darussalam	South-East Asia and developing Pacific
REAS	BTN	Bhutan	Southern Asia
REAS	CHN	China	Eastern Asia
REAS	IDN	Indonesia	South-East Asia and developing Pacific
REAS	IND	India	Southern Asia
REAS	KHM	Cambodia	South-East Asia and developing Pacific
REAS	LAO	Lao People's Democratic Republic	South-East Asia and developing Pacific
REAS	LKA	Sri Lanka	Southern Asia
REAS	MDV	Maldives	Southern Asia
REAS	MMR	Myanmar	South-East Asia and developing Pacific
REAS	MNG	Mongolia	Eastern Asia
REAS	MYS	Malaysia	South-East Asia and developing Pacific
REAS	NPL	Nepal	Southern Asia
REAS	PAK	Pakistan	Southern Asia
REAS	PHL	Philippines	South-East Asia and developing Pacific
REAS	PRK	Korea, Democratic People's Republic of	Eastern Asia

REAS	SGP	Singapore	South-East Asia and developing Pacific
REAS	THA	Thailand	South-East Asia and developing Pacific
REAS	TWN	Taiwan_Province of China	Eastern Asia
REAS	VNM	Viet Nam	South-East Asia and developing Pacific
US EPA	PRI	Puerto Rico	North America
US EPA	USA	United States	North America
US EPA	VIR	Virgin Islands_USA	North America

S2 – Comparison of HTAP_v3 emission mosaic vs. regional and global inventories

In this section, the comparison between the HTAP_v3 mosaic emission time series by pollutant and region and the corresponding emissions from other inventories is presented (Figures S1-S5). In particular, we compare HTAP_v3 against CEDS_v2021_04_21 (O'Rourke, 2021), EDGARv5.0 (https://edgar.jrc.ec.europa.eu/dataset_ap50, (Oreggioni et al., 2022)), EDGARv6.1 (which is used in HTAP_v3 as gapfilling inventory, https://edgar.jrc.ec.europa.eu/dataset_ap61), country inventories, GAINS_(ECLIPSE_v6b_CLE) (Klimont et al., 2017), and REAS_v3.2.1 (Kurokawa and Ohara, 2020) including the latest updates available at https://www.nies.go.jp/REAS/. In a few instances (Canada, China) multiple versions of the country inventories are available and the older version is plotted as "older_country_inv" in the first figure in each set below.

The country level inventories are from the US EPA (https://www.epa.gov/air-emissions-inventories/air-pollutant-emissions-trends-data; Version April 27, 2020), Environment and Climate Change Canada (https://open.canada.ca/data/en/dataset/fa1c88a8-bf78-4fcb-9c1e-2a5534b92131, downloaded August 2020), EMEP (https://www.ceip.at/, downloaded 2020), MEIC for China (Zheng et al 2018), Japan (REAS 3.2.1), Korea (http://airemiss.nier.go.kr/; downloaded 2020) and Taiwan (http://teds.epa.gov.tw/ various databases and documents downloaded in 2020).

For each emission species two graphs are shown. The first shows comparisons at the country level (and Eastern/western European regions) where we can compare HTAP_v3 with the country-level inventory data as processed for CEDS. Note that CEDS is calibrated to the country level data shown in this graph, which means CEDS will generally align with the country-level data except where gap filling has taken place (as noted below). The second graph in each set below shows a comparison at the level of global regions, where we compare the three global inventories and, for Asia regions, REAS.

The graphs below exclude emissions from aviation, international shipping, and agricultural waste burning on fields. Note that, while we have attempted to harmonize geographic and sectoral coverage between the inventories, the correspondence is not always exact and this can lead to spurious differences. There are significant differences, for example, in how different inventories define the category "domestic shipping". We, therefore, focus on larger differences where this potentially impacts interpretation of the HTAP_v3 data.

S2.1- Comparison of SO_2 emissions

Overall, HTAP_v3 compares well with the country level data for SO_2. The largest discrepancy is for Australia. The Australia National Pollutant Inventory (NPI; http://www.npi.gov.au/) provides annual data for point sources. For non-point sources, however, this only provides data for one representative year, which is also not necessarily consistent between states. For this reason, there is no consistent time series data available for total emissions from the NPI. The CEDS emission data uses default estimates for area sources, but calibrates emissions from power plants and industrial sources (for species where point sources dominate, such as SO_2 from metal smelters) to the Australia NPI data. HTAP_v3 is based on EDGAR for Australia, as shown in the graph. While SO_2 values are similar by 2018, the NPI indicates that emissions were much higher than the HTAP_v3 values by 2000.

There is also a small difference between inventories for China. While CEDS is calibrated to MEIC, CEDS also contains bottom-up estimates for metal smelting SO_2 emissions that are not included in MEIC which increases total emissions in the mid to late 2000's. The difference becomes small by the end of the time series as increased penetration of acid production plants and pollution control devices is assumed to have substantially decreased SO_2 emissions from metal smelting.

At a regional level the largest differences between inventories, where the CEDS inventory has higher emissions than EDGAR (and HTAP_v3) or GAINS, are in Latin America from metal smelting (derived from bottom-up mass balance estimates plus some country data in CEDS), and the Middle East, where CEDS has higher emissions from oil and gas operations (derived from OMI satellite measurements). Differences in SO_2 emissions between HTAP_v3 and EDGAR_v6 for the Islands[1] regional grouping is associated with the emissions from Maldives which are provided by the REAS inventory and not by EDGAR.

[1]In this comparison, 'Islands' includes emissions from: Cook Islands, Faeroe Islands, Maldives, Martinique, Niue, French Polynesia, Saint Pierre and Miquelon.

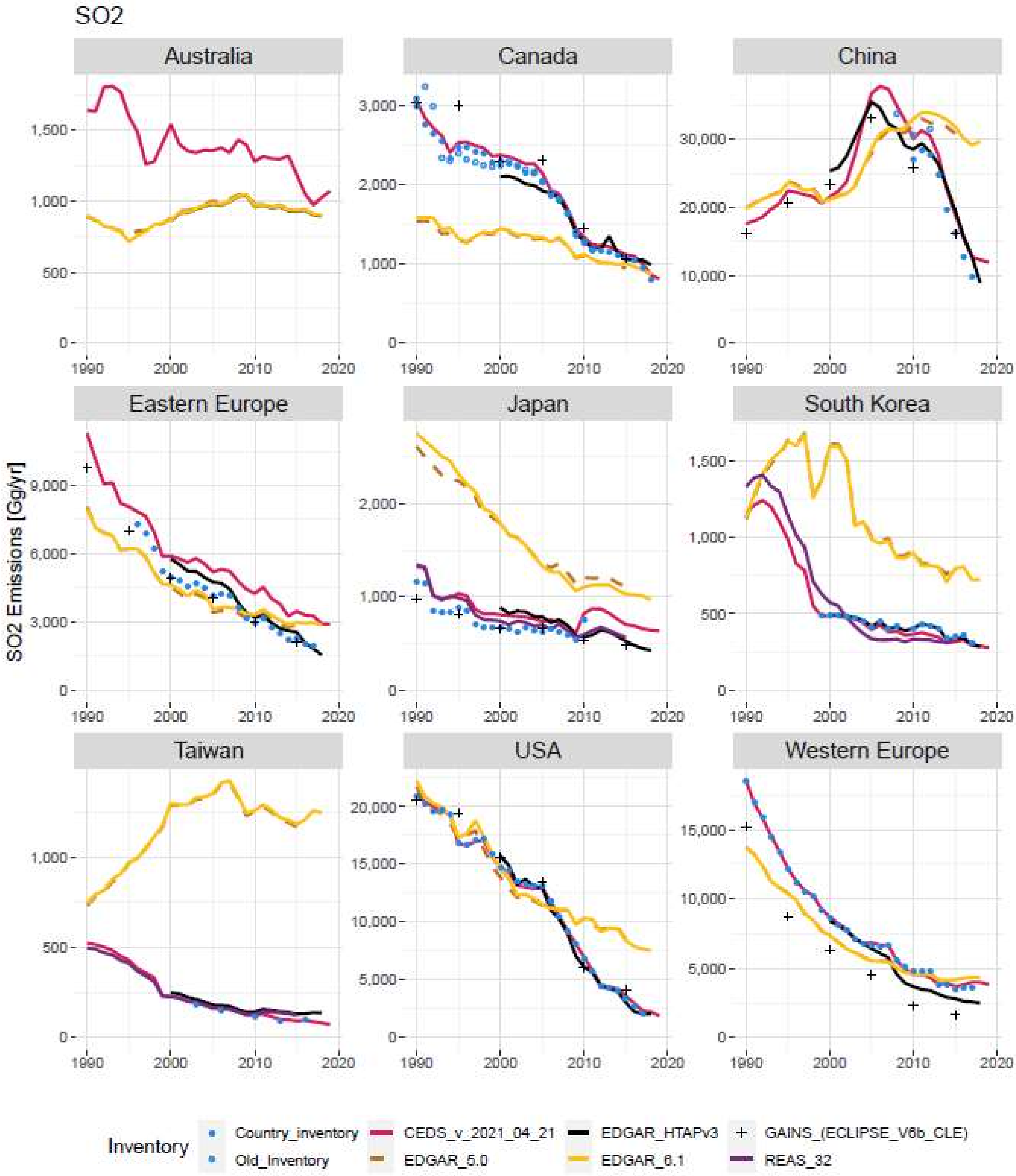
SO2
Australia
Canada
China
Eastern Europe
Japan
South Korea
Taiwan
USA
Western Europe
SO2 Emissions [Gg/yr]
Inventory
Country_inventory
Old_Inventory
CEDS_v_2021_04_21
EDGAR_5.0
EDGAR_HTAPv3
EDGAR_6.1
GAINS_(ECLIPSE_V6b_CLE)
REAS_32

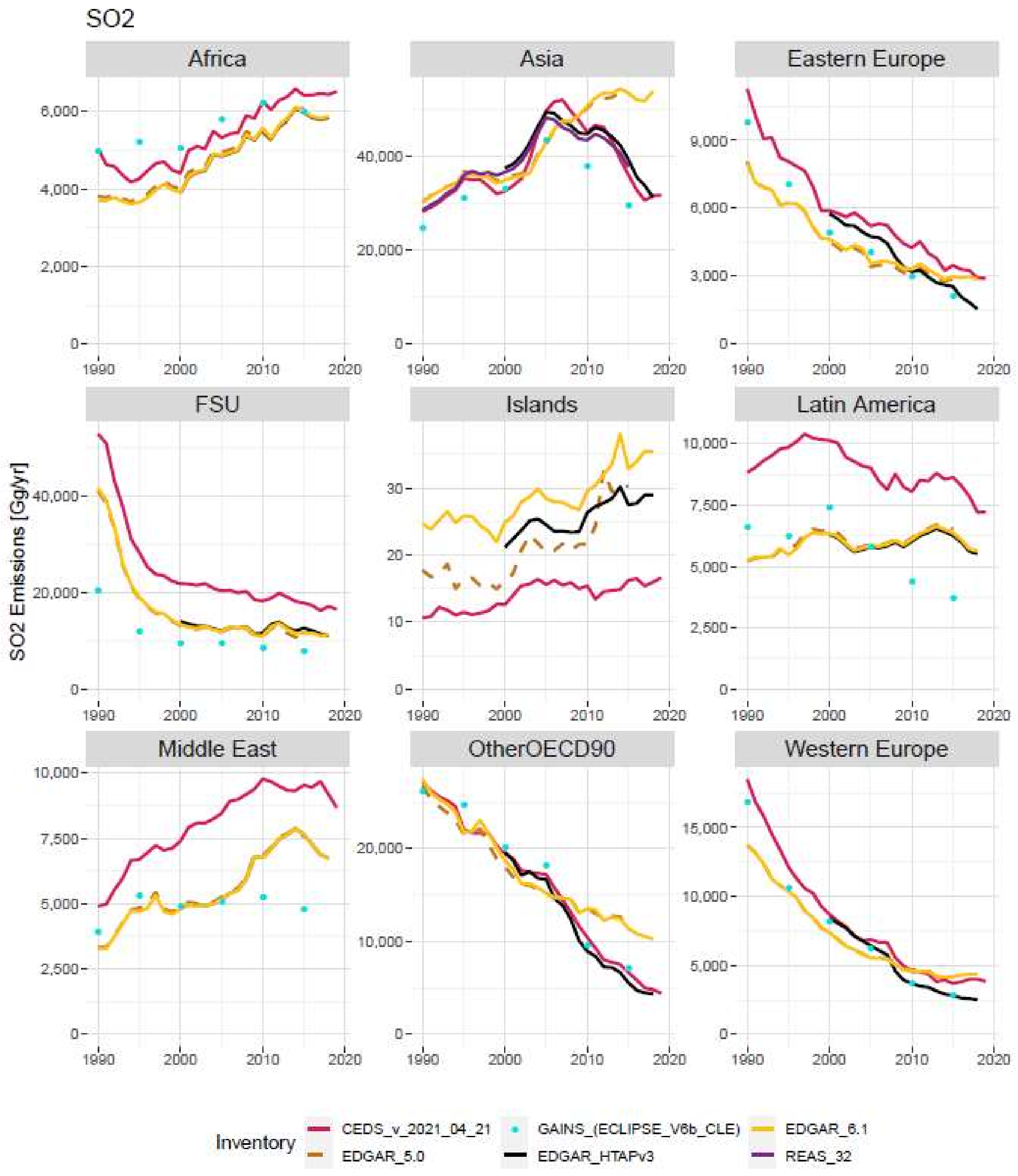

Figure S1 – SO_2 emission time series comparison by world region as provided by different inventories and HTAP_v3.

S2.2- Comparison of NOx emissions

Overall, HTAP_v3 compares well with the country level data for NOx with general agreement between the inventories for most regions. Trends in Australia are particularly uncertain, however, since there is no country-level time series information for mobile sources, which are a major driver of trends in NOx emissions. The figure illustrates how different assumptions impact the estimated trends, which differ between EDGAR v5, EDGAR v6 (on which

HTAP_v3 is based), and CEDS (which uses emission factors from GAINS for mobile sources). The inventories also show different emission magnitudes for Japan. Possible drivers of differences include different assumptions about industrial sector emissions and the treatment of domestic shipping emissions, which are both significant contributors to emission totals in Japan. Similarly to So2, also differences in NOx emissions between HTAP_v3 and EDGAR_v6 for the Islands regional grouping is associated with the emissions from Maldives which are provided by the REAS inventory and not by EDGAR.

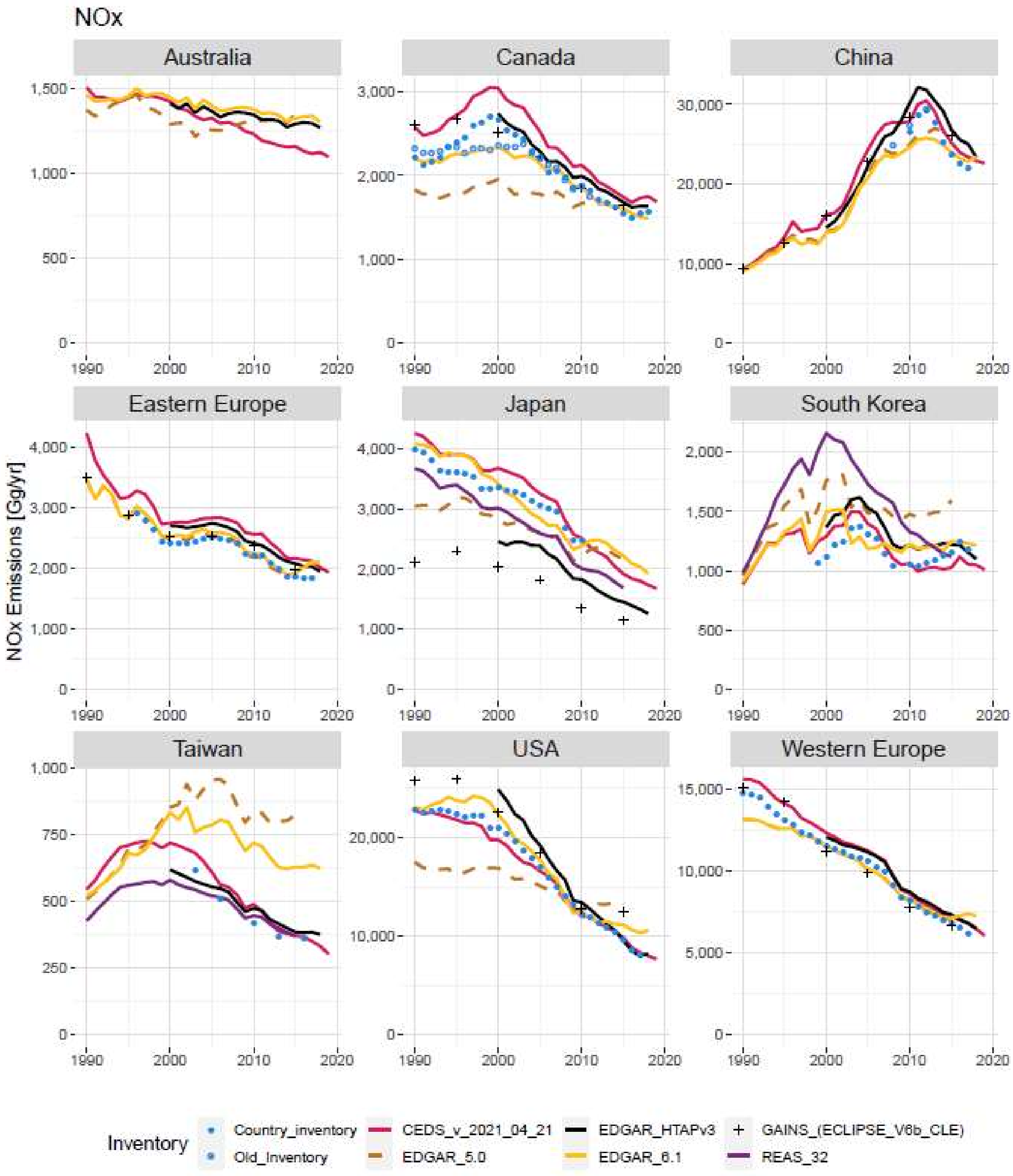

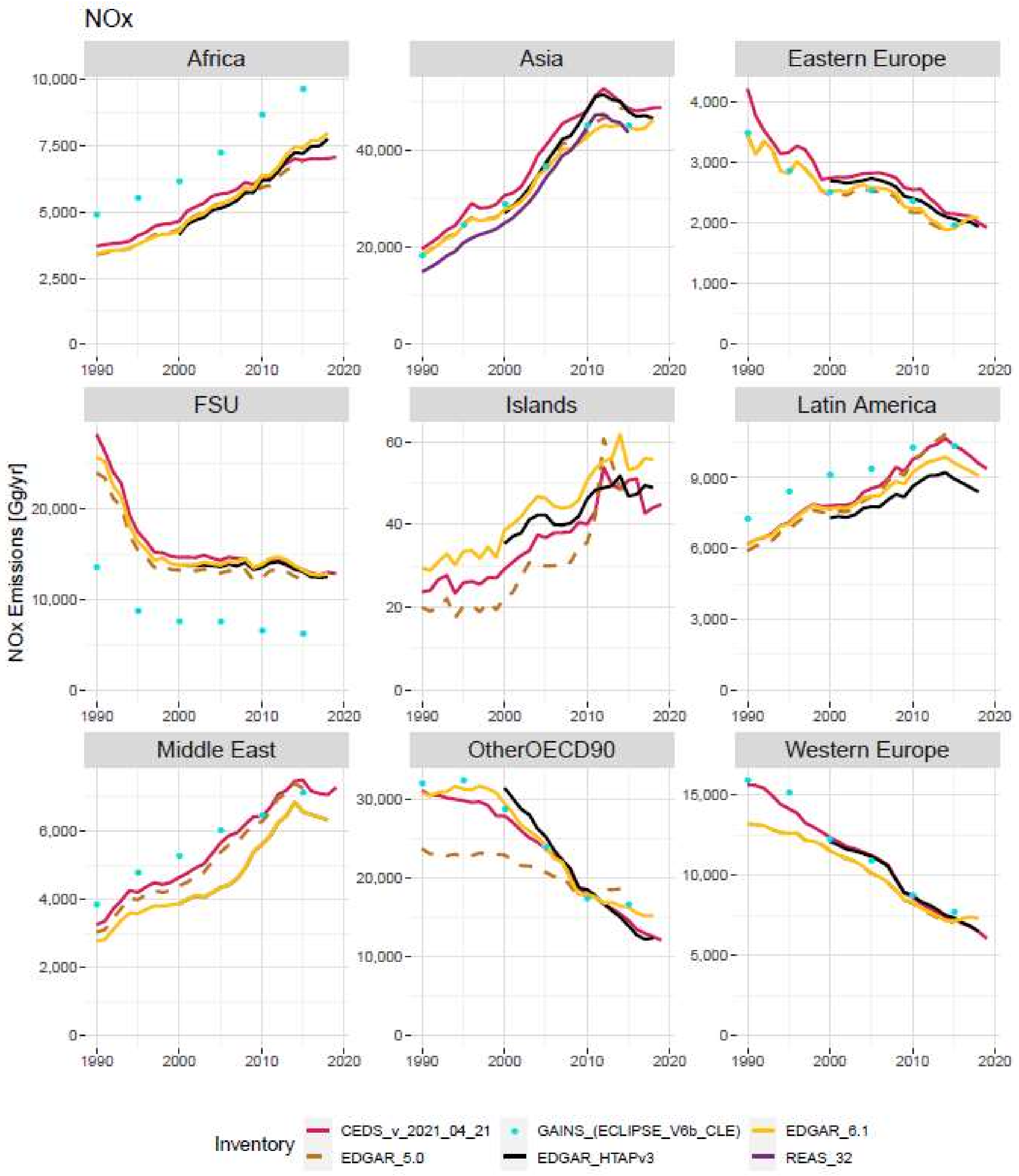

Figure S2 – NOx emission time series comparison by world region as provided by different inventories and HTAP_v3.

S2.3- Comparison of CO emissions

While HTAP_v3 compares well with the country level data for CO, there is a large variation overall between the different global inventories. CO emission factors depend heavily on combustion process details which are difficult to capture with default regional emission factor

assumptions. An example of this is shown for Canada, where CO emission estimates appear to have changed significantly in more recent versions of the inventory.

There is a particularly large difference in China by 2000, where HTAP_v3 has CO emissions that peak around 2010, whereas CEDS has CO emissions peaking earlier and at a higher value, and EDGAR has no prominent peak in CO emissions. The difference is largely in the residential sector, with HTAP_v3 residential CO emissions decreasing when going back from 2005 to 2000, whereas residential sector CO emissions increase in CEDS driven by increases in residential biomass combustion when going back from 2005 to 2000.

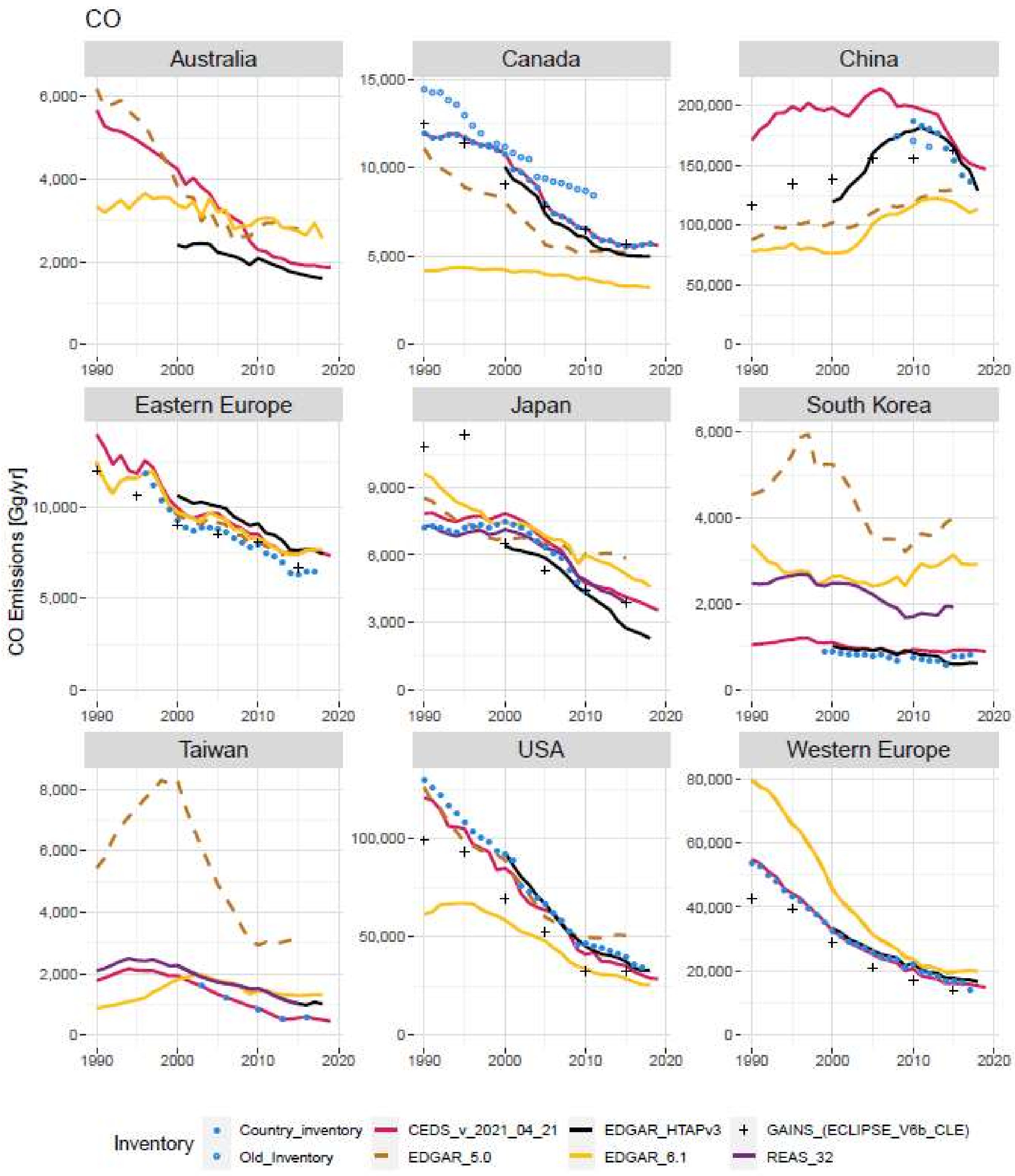

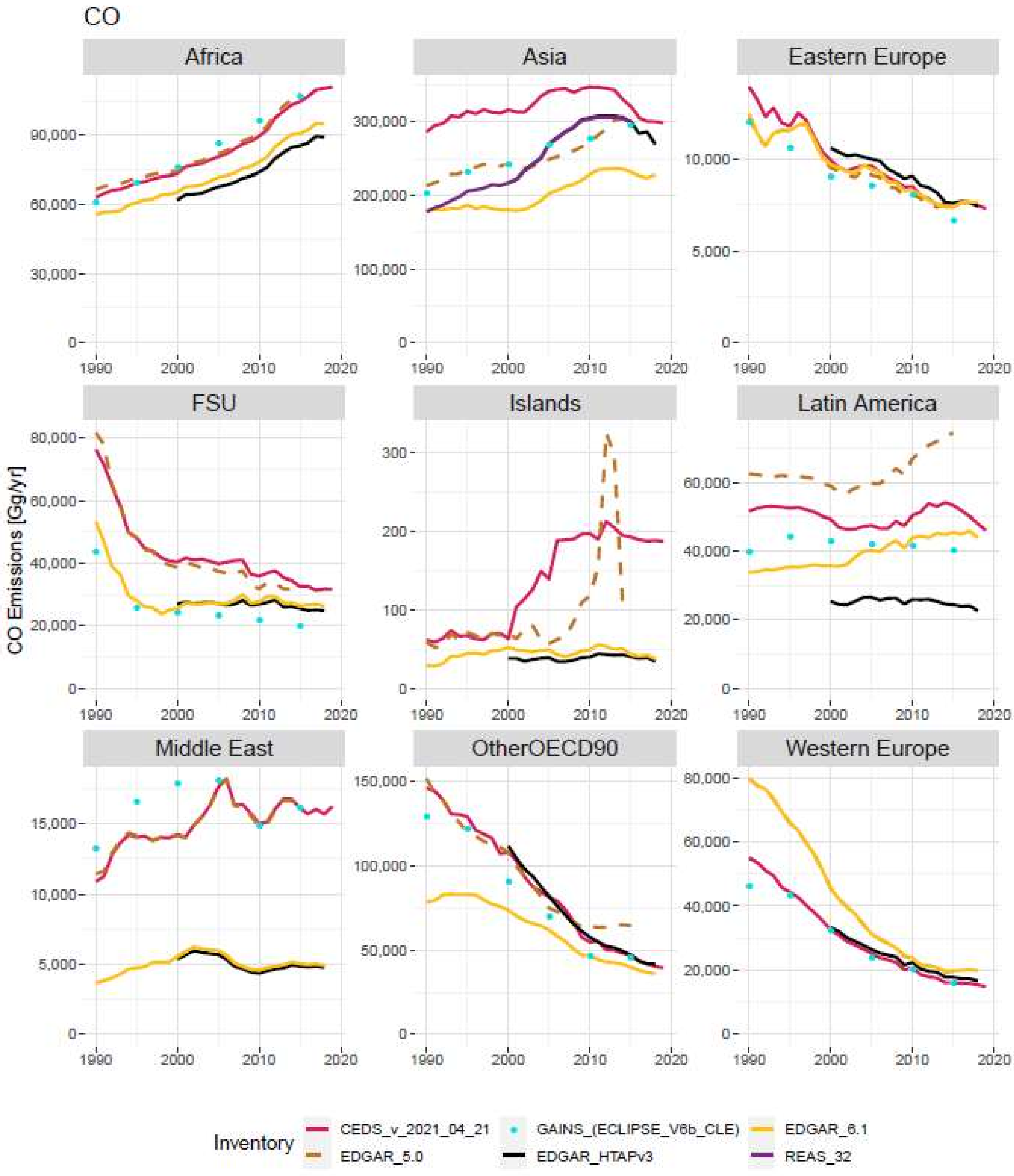

Figure S3 – CO emission time series comparison by world region as provided by different inventories and HTAP_v3.

S2.4- Comparison of NMVOC emissions

While HTAP_v3 compares well with the country level data for NMVOC, there is a large variation overall between the different global inventories. HTAP_v3 NMVOC emissions are shifted higher than the country level inventories used in CEDS for a number of countries/regions. The reason for this difference is not clear, but may be due to more recent country data used in EDGAR-HTAP_v3 or differences in sectoral coverage.

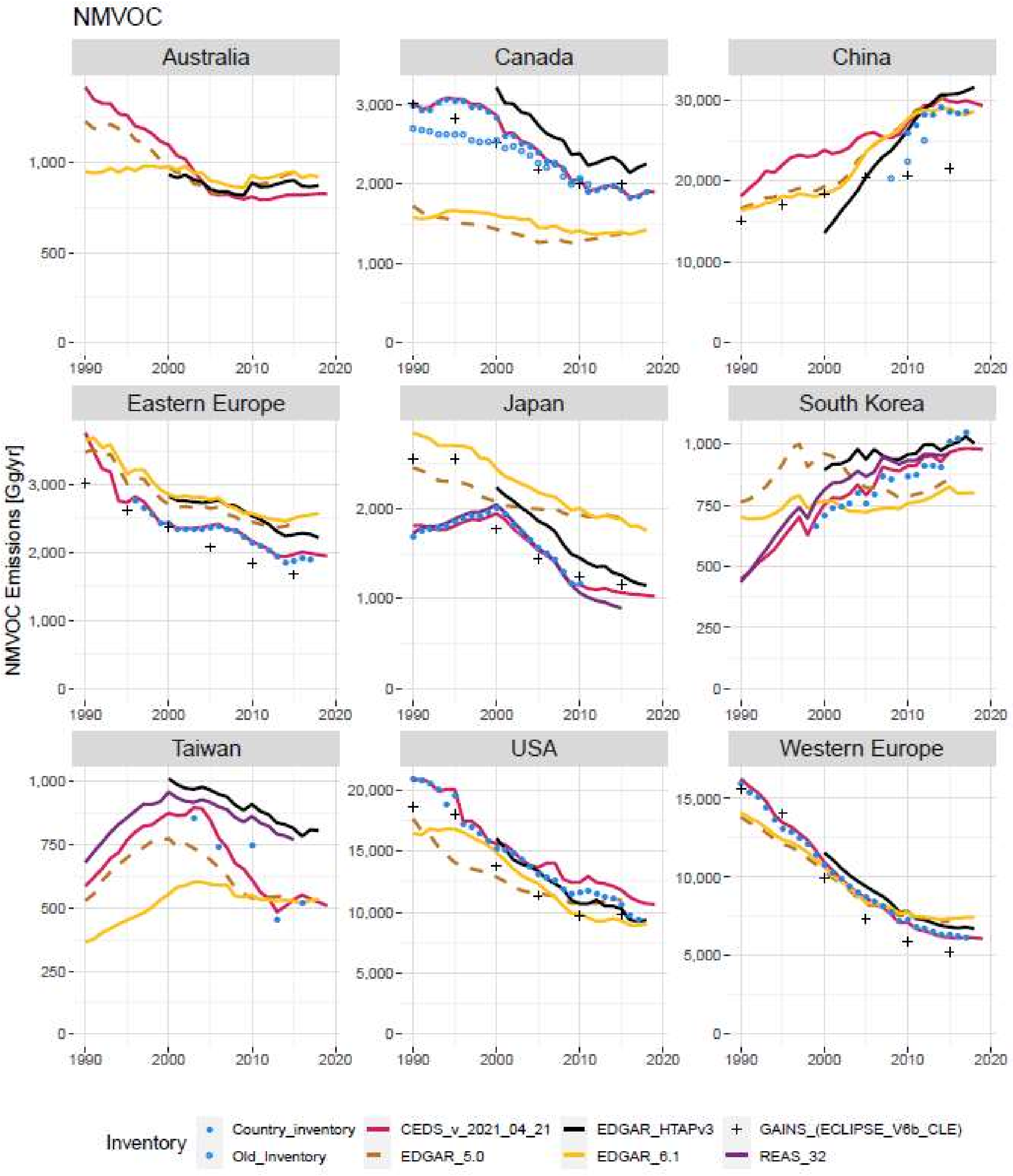
NMVOC
Australia
Canada
China
Eastern Europe
Japan
South Korea
Taiwan
USA
Western Europe
NMVOC Emissions [Gg/yr]
Inventory
Country_inventory
Old_Inventory
CEDS_v_2021_04_21
EDGAR_5.0
EDGAR_HTAPv3
EDGAR_6.1
GAINS_(ECLIPSE_V6b_CLE)
REAS_32

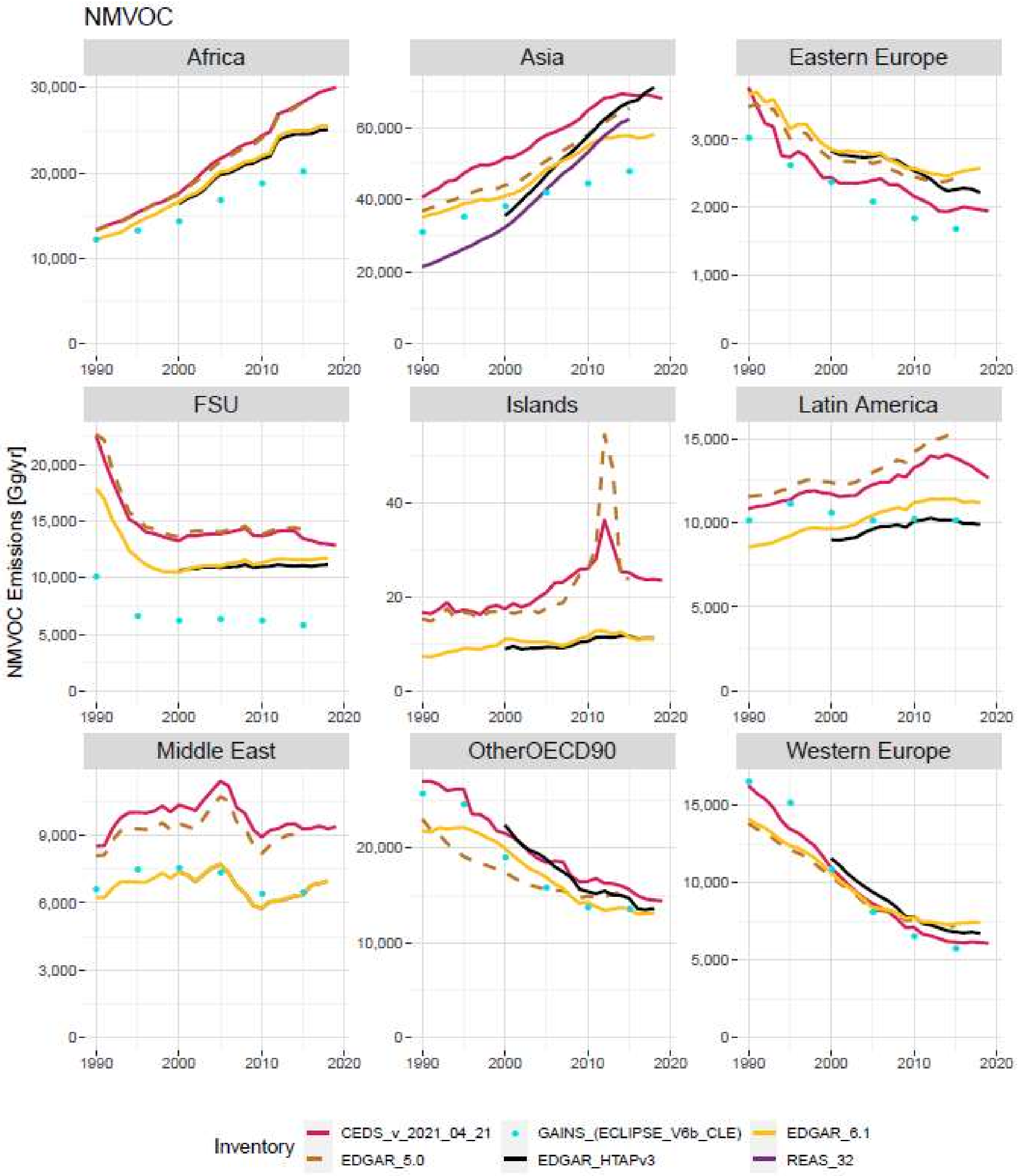

Figure S4 – NMVOC emission time series comparison by world region as provided by different inventories and HTAP_v3.

S2.5- Comparison of NH_3 emissions

While HTAP_v3 compares well with the country level data for NH_3 in most cases, there is also a large variation overall between the different global inventories. In some cases, such as the USA, gridded NH_3 emissions in some key agricultural sectors was not available so these emissions were gap filled from EDGAR estimates.

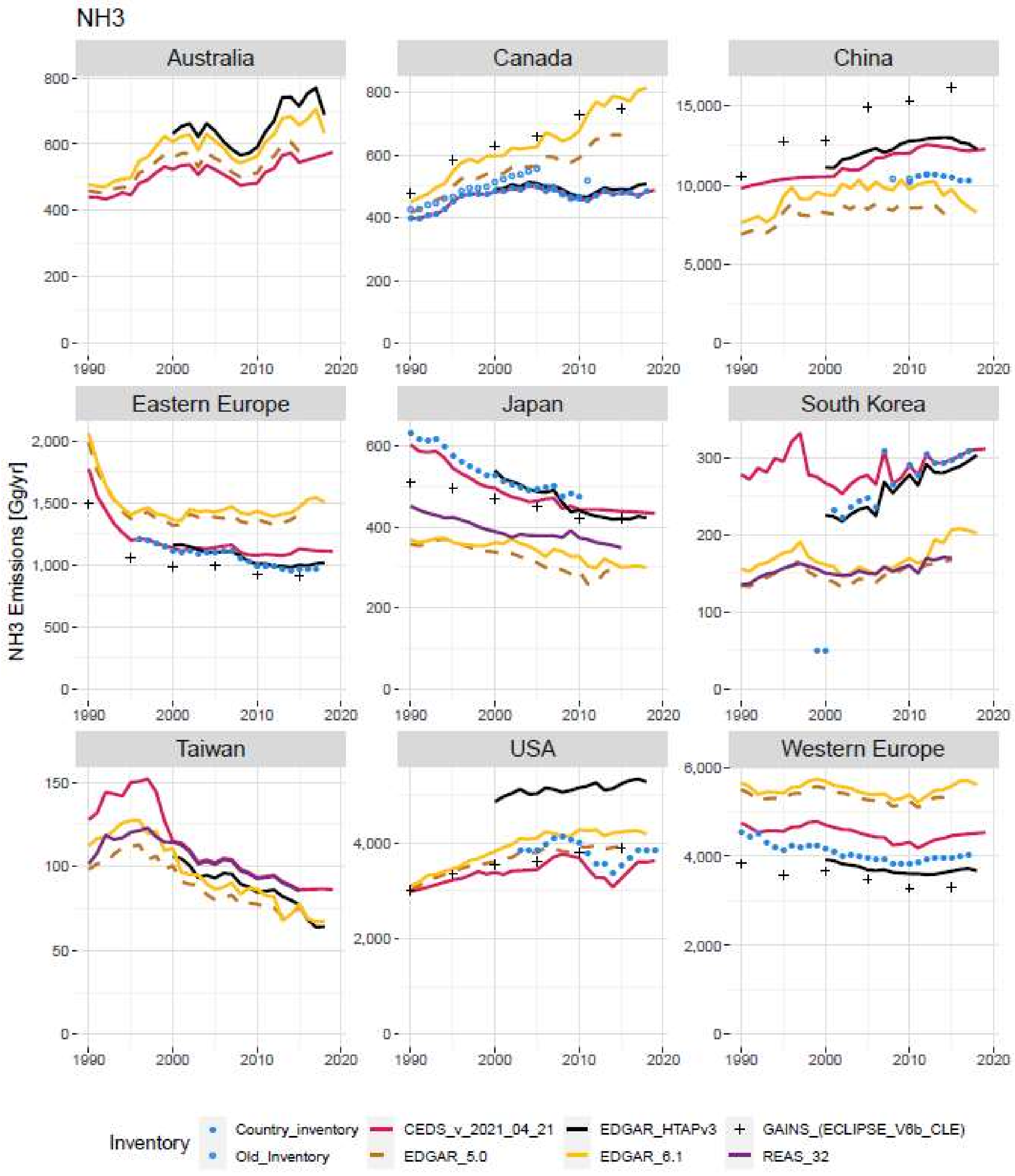

NH3
Australia
Canada
China
Eastern Europe
Japan
South Korea
Taiwan
USA
Western Europe
NH3 Emissions [Gg/yr]
Inventory
Country_inventory
Old_Inventory
CEDS_v_2021_04_21
EDGAR_5.0
EDGAR_HTAPv3
EDGAR_6.1
GAINS_(ECLIPSE_V6b_CLE)
REAS_32

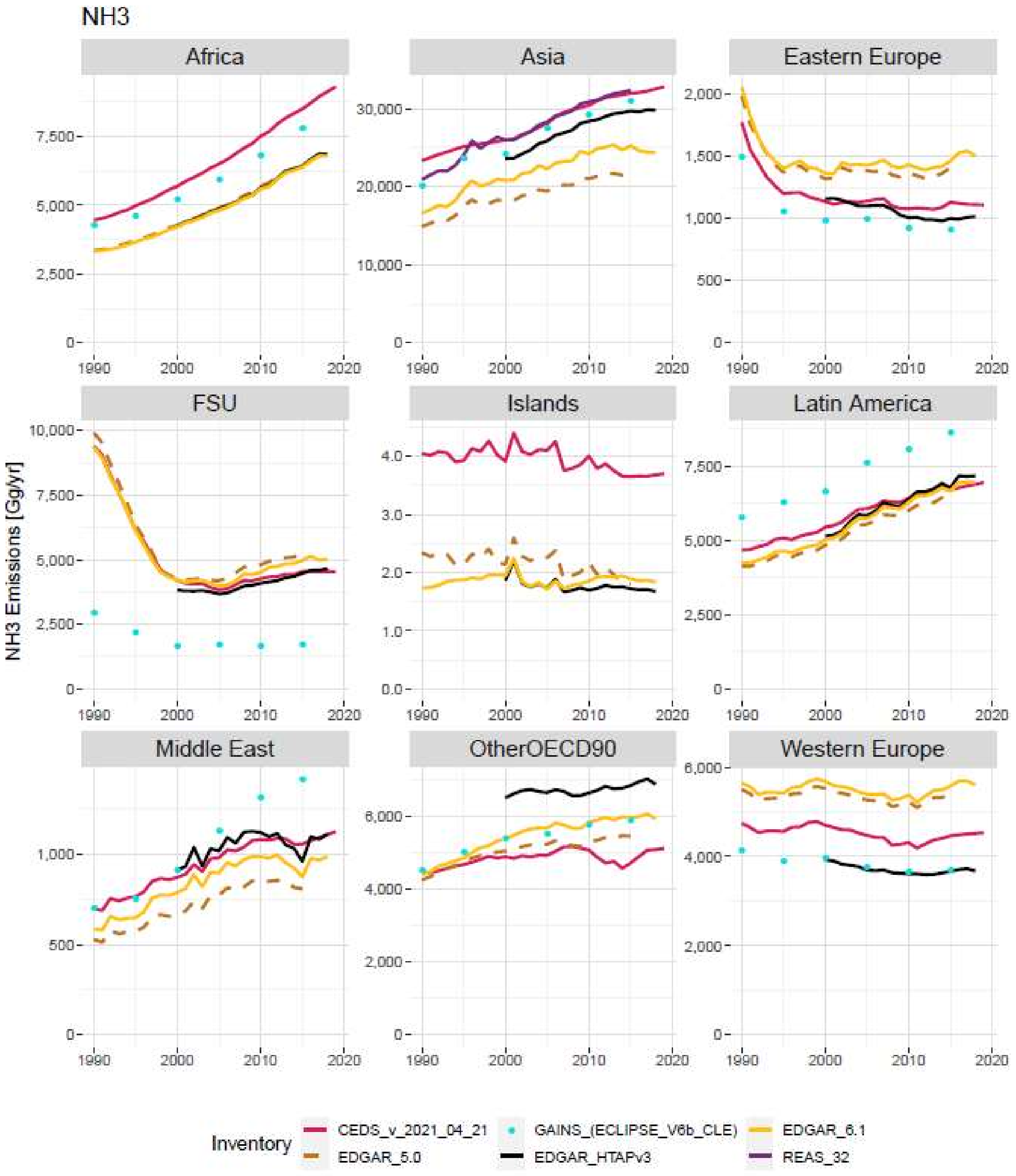

Figure S5 – NH_3 emission time series comparison by world region as provided by different inventories and HTAP_v3.

S3 – Monthly variability of the emissions

Figures S6, S7 and S8 show the monthly contribution of the emissions of CO, NMVOC and SO_2 in 2015 for world regions. The largest variability is found for the residential sector and agriculture, while smaller variation is present for energy, industry and transport.

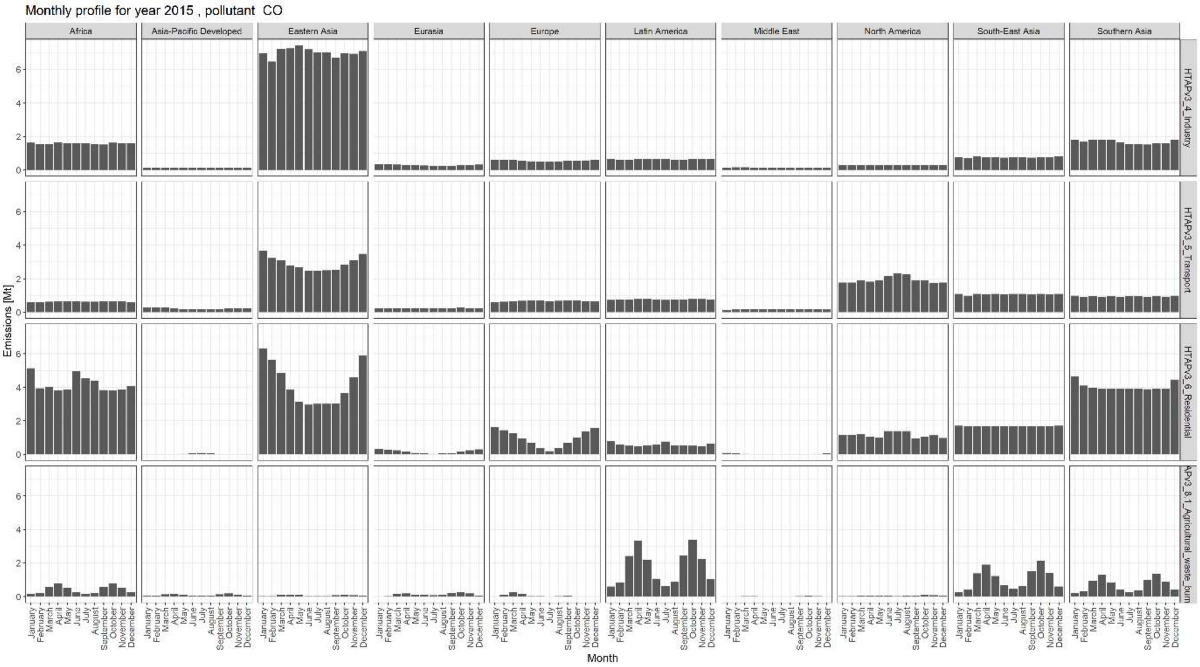

Figure S6 – Monthly variability of CO emissions for relevant emission sectors for the different world regions in 2015.

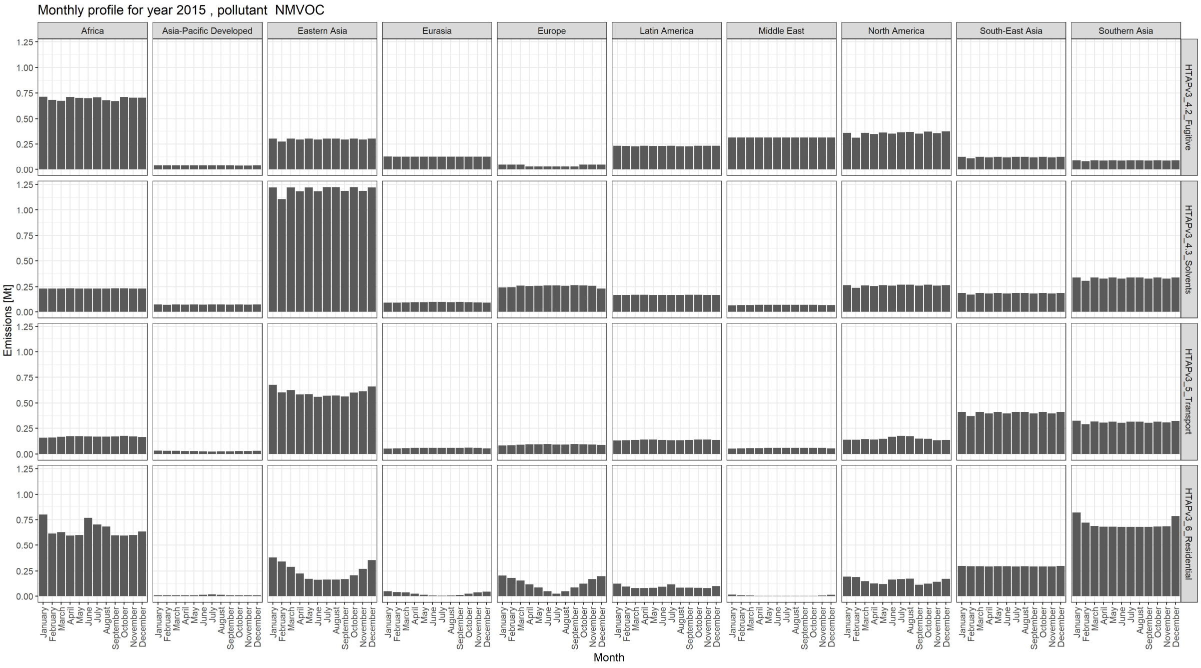

Figure S7 – Monthly variability of NMVOC emissions for relevant emission sectors for the different world regions in 2015.

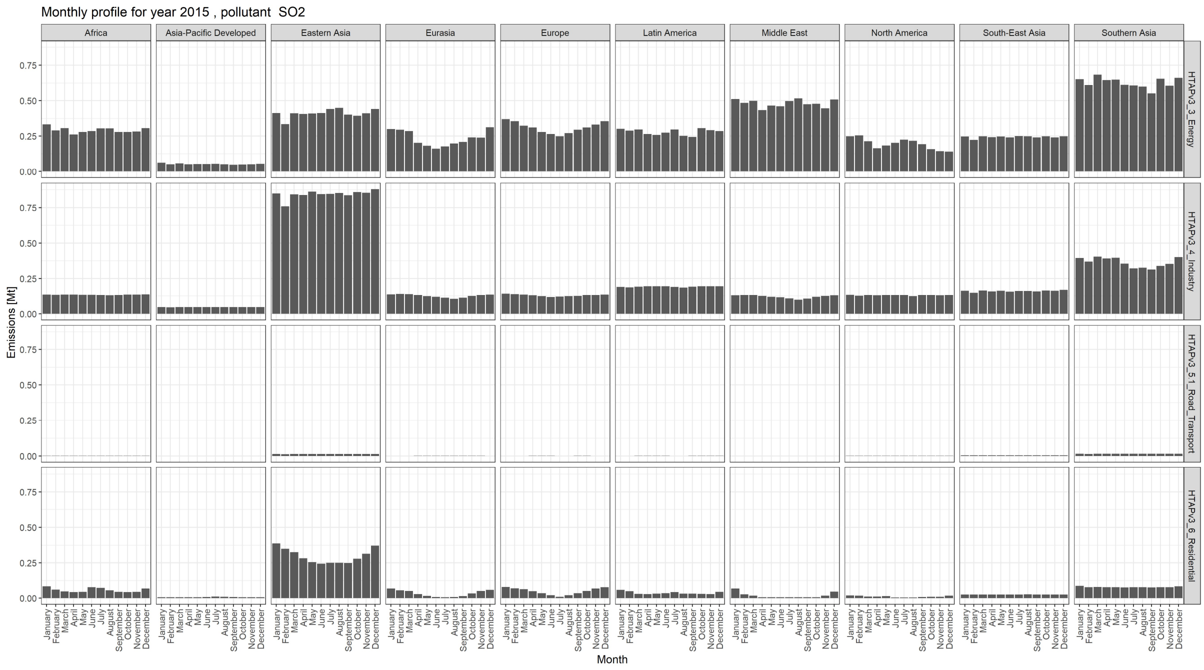

Figure S8 – Monthly variability of SO_2 emissions for relevant emission sectors for the different world regions in 2015.

Figures S9-S14 show the monthly mean profiles for the different pollutants and relevant emission sectors. The mean profile over the years 2002-2015, which is the period covered by all data providers, for each region is represented together with the 10° and 90° percentiles. With the exception of few data providers (EDGAR and ECCC), no inter-annual variability of the monthly profiles is found.

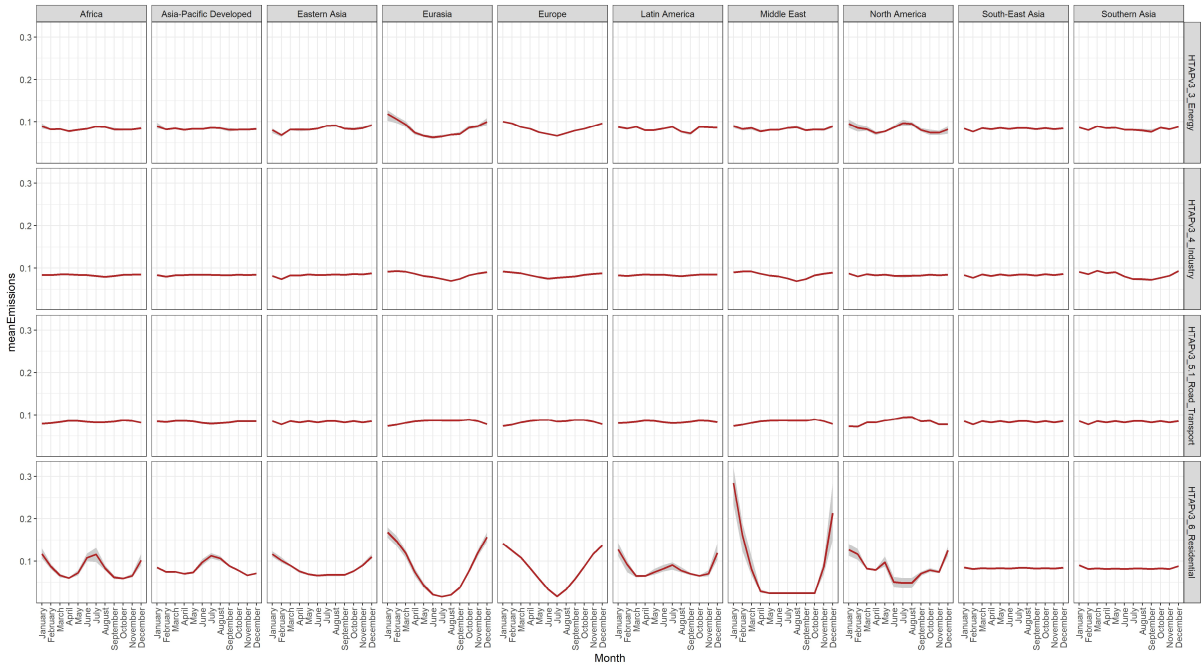

Figure S9 – Monthly mean profile of SO_2 emissions for relevant emission sectors for the different world regions. The mean profile over the years 2002-2015, which is the period

covered by all data providers, for each region is represented together with the 10° and 90° percentiles, in grey.

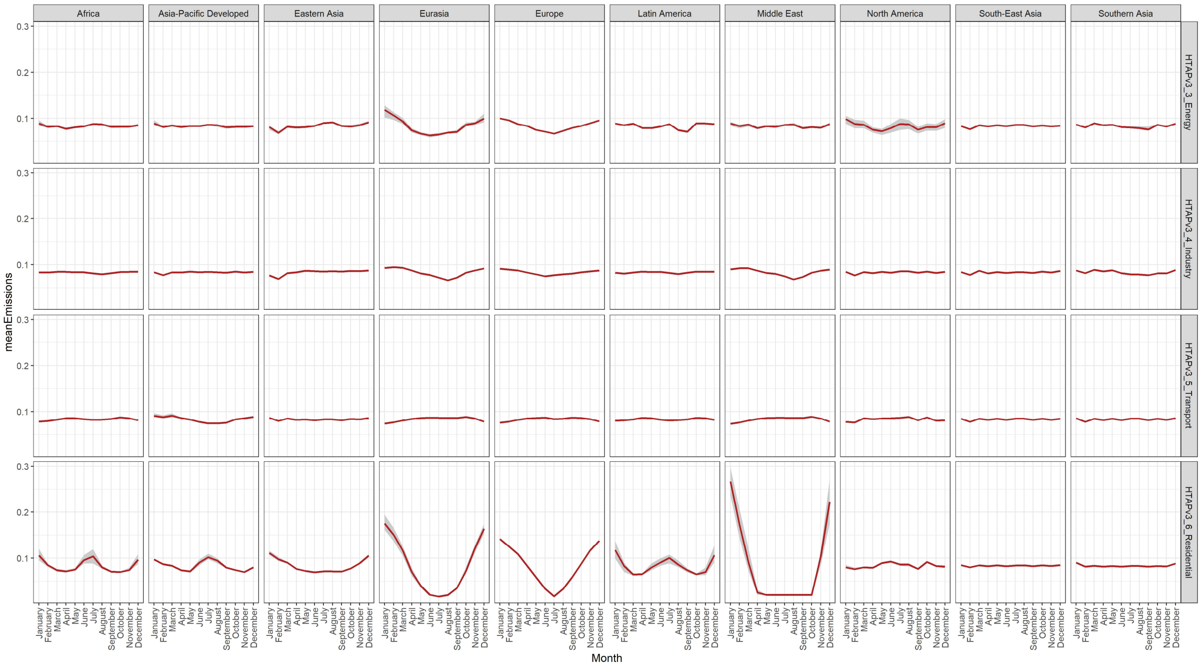

Figure S10 – Monthly mean profile of NOx emissions for relevant emission sectors for the different world regions. The mean profile over the years 2002-2015, which is the period covered by all data providers, for each region is represented together with the 10° and 90° percentiles.

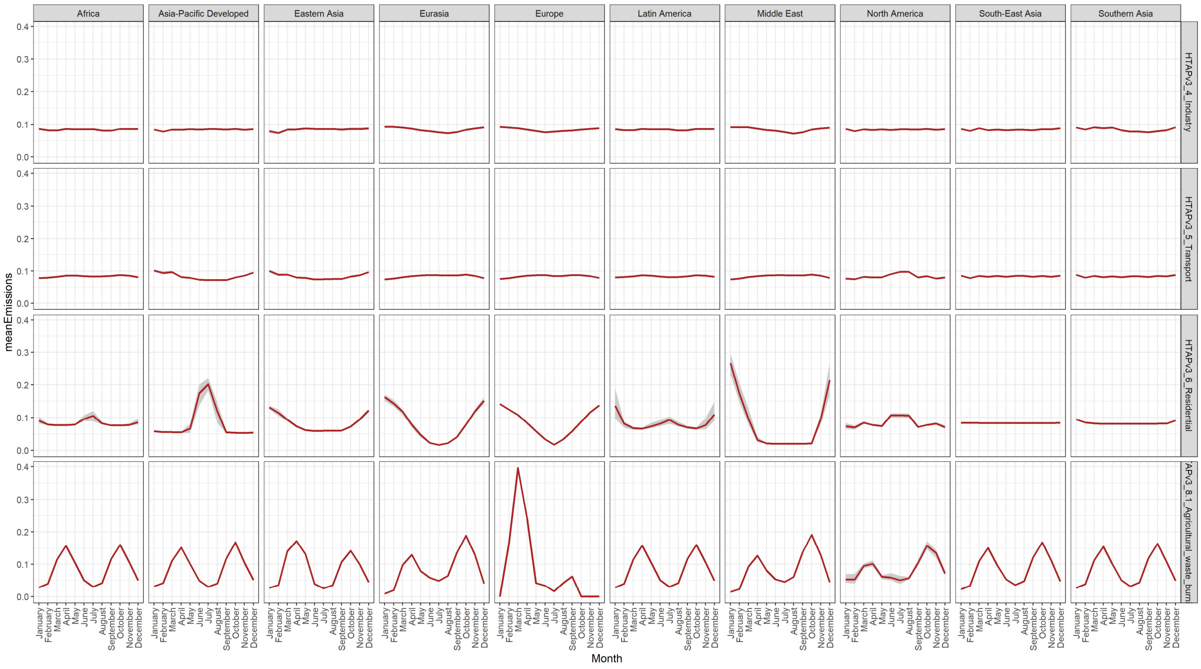

Figure S11 – Monthly mean profile of CO emissions for relevant emission sectors for the different world regions. The mean profile over the years 2002-2015, which is the period covered by all data providers, for each region is represented together with the 10° and 90° percentiles.

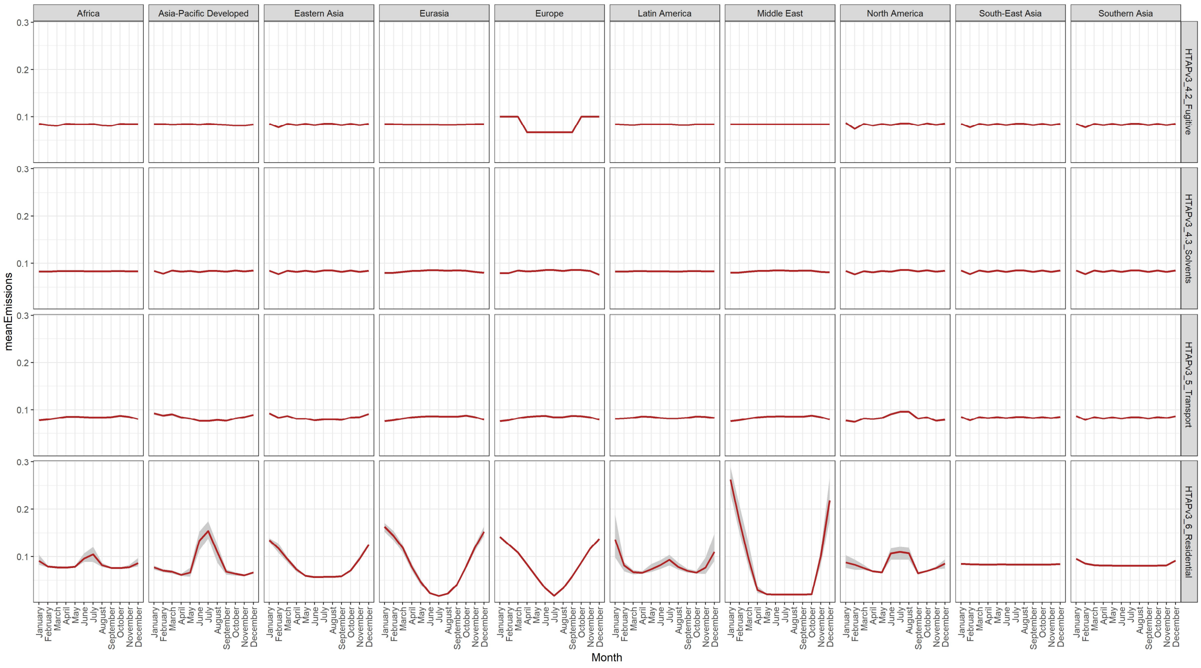

Figure S12 – Monthly mean profile of NMVOC emissions for relevant emission sectors for the different world regions. The mean profile over the years 2002-2015, which is the period covered by all data providers, for each region is represented together with the 10° and 90° percentiles.

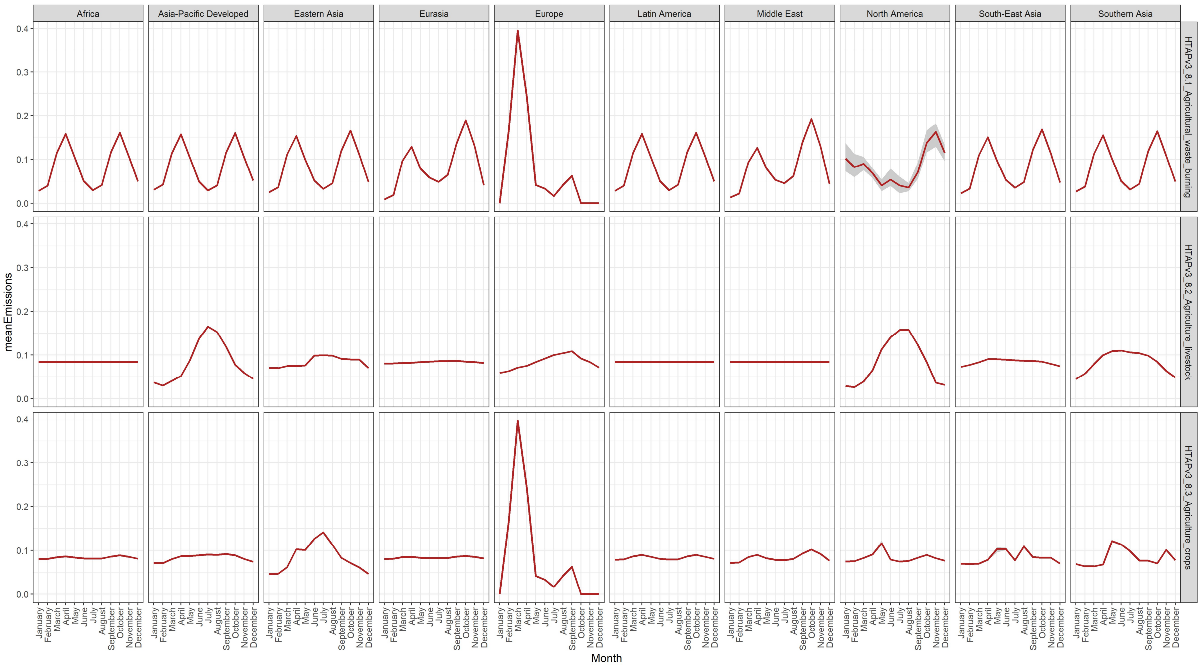

Figure S13 – Monthly mean profile of NH_3 emissions for relevant emission sectors for the different world regions. The mean profile over the years 2002-2015, which is the period covered by all data providers, for each region is represented together with the 10° and 90° percentiles.

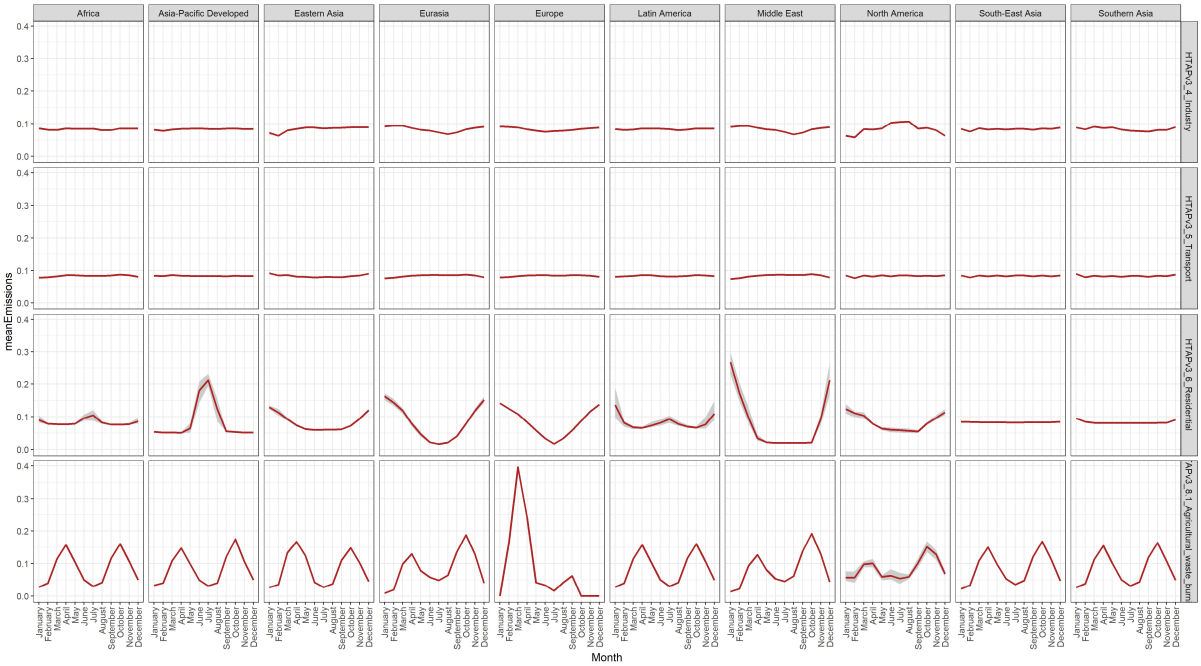

Figure S14 – Monthly mean profile of PM_{10} emissions for relevant emission sectors for the different world regions. The mean profile over the years 2002-2015, which is the period covered by all data providers, for each region is represented together with the 10° and 90° percentiles.

S4 NMVOC speciation

Table S4 provides the list of Global Emissions InitiAtive (GEIA) 25 NMVOC groups included in HTAP_v3 with the corresponding molecular formula.

Table S4 – List of NMVOC species included in HTAP_v3. R and R' denote functional groups. Where general formulae are not appropriate, the simplest molecular formula representing the group is provided. NA = not available

GEIA ID	GEIA group	Molecular formula
voc1	Alkanols (alcohols)	$C_nH_{2n}+_1OH$
voc2	Ethane	C_2H_6
voc3	Propane	C_3H_8
voc4	Butanes	C_4H_{10}
voc5	Pentanes	C_5H_{12}
voc6	Hexanes and higher alkanes	C_nH_{2n+2} ($n \geq 6$)
voc7	Ethene (ethylene)	C_2H_4
voc8	Propene	C_3H_6
voc9	Ethyne (acetylene)	C_2H_2
voc10	Isoprenes	C_5H_8
voc11	Monoterpenes	$C_{10}H_{16}$

voc12	Other alk(adi)enes/alkynes (olefines)	C_nH_{2n-2}
voc13	Benzene (benzol)	C_6H_6
voc14	Methylbenzene (toluene)	C_7H_8
voc15	Dimethylbenzenes (xylenes)	$C_6H_4(CH_3)_2$
voc16	Trimethylbenzenes	$C_6H_3(CH_3)_3$
voc17	Other aromatics	C_nH_{2n-6}
voc18	Esters	R-C(=O)O-R'
voc19	Ethers (alkoxy alkanes)	R-O-R'
voc20	Chlorinated hydrocarbons	CH_3Cl
voc21	Methanal (formaldehyde)	CH_2O
voc22	Other alkanals (aldehyedes)	R-CHO
voc23	Alkanones (ketones)	R-C(=O)-R'
voc24	Acids (alkanoic)	R-C_nH_nCOOH
voc25	Other NMVOC (HCFCs, nitriles, etc.)	NA

Table S5 - Regional mapping to be applied for NMVOC speciation

Country code	Country name	Regionala_VOC_grouping	Region definition
ABW	Aruba	OT	Other
AFG	Afghanistan	AS	Asia
AGO	Angola	OT	Other
AIA	Anguilla	OT	Other
AIR	Int. Aviation	OT	Other
ALA	Åland Islands	EU	Europe
ALB	Albania	EU	Europe
AND	Andorra	EU	Europe
ANT	Netherlands Antilles	OT	Other
ARE	United Arab Emirates	OT	Other
ARG	Argentina	OT	Other
ARM	Armenia	EU	Europe
ASM	American Samoa	OT	Other
ATA	Antarctica	OT	Other
ATF	French Southern Territories	OT	Other
ATG	Antigua and Barbuda	OT	Other
AUS	Australia	OT	Other
AUT	Austria	EU	Europe
AZE	Azerbaijan	EU	Europe
BDI	Burundi	OT	Other
BEL	Belgium	EU	Europe
BEN	Benin	OT	Other

BFA	Burkina Faso	OT	Other
BGD	Bangladesh	AS	Asia
BGR	Bulgaria	EU	Europe
BHR	Bahrain	OT	Other
BHS	Bahamas	OT	Other
BIH	Bosnia and Herzegovina	EU	Europe
BLR	Belarus	EU	Europe
BLZ	Belize	OT	Other
BMU	Bermuda	OT	Other
BOL	Bolivia	OT	Other
BRA	Brazil	OT	Other
BRB	Barbados	OT	Other
BRN	Brunei Darussalam	AS	Asia
BTN	Bhutan	AS	Asia
BVT	Bouvet Island	OT	Other
BWA	Botswana	OT	Other
CAF	Central African Republic	OT	Other
CAN	Canada	NA	North America
CCK	Cocos (Keeling) Islands	OT	Other
CHE	Switzerland	EU	Europe
CHL	Chile	OT	Other
CHN	China	AS	Asia
CIV	Cote d'Ivoire	OT	Other
CMR	Cameroon	OT	Other
COD	Congo_the Democratic Republic of the	OT	Other
COG	Congo	OT	Other
COK	Cook Islands	OT	Other
COL	Colombia	OT	Other
COM	Comoros	OT	Other
CPV	Cape Verde	OT	Other
CRI	Costa Rica	OT	Other
CUB	Cuba	OT	Other
CXR	Christmas Island	OT	Other
CYM	Cayman Islands	OT	Other
CYP	Cyprus	EU	Europe
CZE	Czech Republic	EU	Europe
DEU	Germany	EU	Europe
DJI	Djibouti	OT	Other
DMA	Dominica	OT	Other
DNK	Denmark	EU	Europe
DOM	Dominican Republic	OT	Other
DZA	Algeria	OT	Other
E27	Europe - 27 MS	EU	Europe
ECU	Ecuador	OT	Other
EGY	Egypt	OT	Other
ERI	Eritrea	OT	Other

ESH	Western Sahara	OT	Other
ESP	Spain	EU	Europe
EST	Estonia	EU	Europe
ETH	Ethiopia	OT	Other
FIN	Finland	EU	Europe
FJI	Fiji	OT	Other
FLK	Falkland Islands (Malvinas)	OT	Other
FRA	France	EU	Europe
FRO	Faroe Islands	EU	Europe
FSM	Micronesia, Federated States of	OT	Other
GAB	Gabon	OT	Other
GBR	United Kingdom	EU	Europe
GEO	Georgia	EU	Europe
GGY	Guernsey	EU	Europe
GHA	Ghana	OT	Other
GIB	Gibraltar	EU	Europe
GIN	Guinea	OT	Other
GLP	Guadeloupe	OT	Other
GMB	Gambia	OT	Other
GNB	Guinea-Bissau	OT	Other
GNQ	Equatorial Guinea	OT	Other
GRC	Greece	EU	Europe
GRD	Grenada	OT	Other
GRL	Greenland	EU	Europe
GTM	Guatemala	OT	Other
GUF	French Guiana	OT	Other
GUM	Guam	OT	Other
GUY	Guyana	OT	Other
HKG	Hong Kong	AS	Asia
HMD	Heard Island and McDonald Islands	OT	Other
HND	Honduras	OT	Other
HRV	Croatia	EU	Europe
HTI	Haiti	OT	Other
HUN	Hungary	EU	Europe
IDN	Indonesia	AS	Asia
IMN	Isle of Man	EU	Europe
IND	India	AS	Asia
IOT	British Indian Ocean Territory	AS	Asia
IRL	Ireland	EU	Europe
IRN	Iran, Islamic Republic of	OT	Other
IRQ	Iraq	OT	Other
ISL	Iceland	EU	Europe
ISR	Israel	OT	Other
ITA	Italy	EU	Europe
JAM	Jamaica	OT	Other
JEY	Jersey	EU	Europe

JOR	Jordan	OT	Other
JPN	Japan	AS	Asia
KAZ	Kazakhstan	AS	Asia
KEN	Kenya	OT	Other
KGZ	Kyrgyzstan	AS	Asia
KHM	Cambodia	AS	Asia
KIR	Kiribati	OT	Other
KNA	Saint Kitts and Nevis	OT	Other
KOR	Korea, Republic of	AS	Asia
KWT	Kuwait	OT	Other
LAO	Lao People's Democratic Republic	AS	Asia
LBN	Lebanon	OT	Other
LBR	Liberia	OT	Other
LBY	Libyan Arab Jamahiriya	OT	Other
LCA	Saint Lucia	OT	Other
LIE	Liechtenstein	EU	Europe
LKA	Sri Lanka	AS	Asia
LSO	Lesotho	OT	Other
LTU	Lithuania	EU	Europe
LUX	Luxembourg	EU	Europe
LVA	Latvia	EU	Europe
MAC	Macao	AS	Asia
MAR	Morocco	OT	Other
MCO	Monaco	EU	Europe
MDA	Moldova, Republic of	EU	Europe
MDG	Madagascar	OT	Other
MDV	Maldives	AS	Asia
MEX	Mexico	OT	Other
MHL	Marshall Islands	OT	Other
MKD	Macedonia, the former Yugoslav Republic of	EU	Europe
MLI	Mali	OT	Other
MLT	Malta	EU	Europe
MMR	Myanmar	AS	Asia
MNE	Montenegro	EU	Europe
MNG	Mongolia	AS	Asia
MNP	Northern Mariana Islands	OT	Other
MOZ	Mozambique	OT	Other
MRT	Mauritania	OT	Other
MSR	Montserrat	OT	Other
MTQ	Martinique	OT	Other
MUS	Mauritius	OT	Other
MWI	Malawi	OT	Other
MYS	Malaysia	AS	Asia
MYT	Mayotte	OT	Other
NAM	Namibia	OT	Other

NCL	New Caledonia	OT	Other
NER	Niger	OT	Other
NFK	Norfolk Island	OT	Other
NGA	Nigeria	OT	Other
NIC	Nicaragua	OT	Other
NIU	Niue	OT	Other
NLD	Netherlands	EU	Europe
NOR	Norway	EU	Europe
NPL	Nepal	AS	Asia
NRU	Nauru	OT	Other
NZL	New Zealand	OT	Other
OMN	Oman	OT	Other
PAK	Pakistan	AS	Asia
PAN	Panama	OT	Other
PCN	Pitcairn	OT	Other
PER	Peru	OT	Other
PHL	Philippines	AS	Asia
PLW	Palau	OT	Other
PNG	Papua New Guinea	AS	Asia
POL	Poland	EU	Europe
PRI	Puerto Rico	OT	Other
PRK	Korea, Democratic People's Republic of	AS	Asia
PRT	Portugal	EU	Europe
PRY	Paraguay	OT	Other
PSE	Palestinian Territory	OT	Other
PYF	French Polynesia	OT	Other
QAT	Qatar	OT	Other
REU	Reunion	OT	Other
ROU	Romania	EU	Europe
RUS	Russian Federation	EU	Europe
RWA	Rwanda	OT	Other
SAU	Saudi Arabia	OT	Other
SCG	Serbia and Montenegro	EU	Europe
SDN	Sudan	OT	Other
SEA	Int. Shipping	OT	Other
SEN	Senegal	OT	Other
SGP	Singapore	AS	Asia
SGS	South Georgia and the South Sandwich Islands	OT	Other
SHN	Saint Helena	OT	Other
SJM	Svalbard and Jan Mayen	EU	Europe
SLB	Solomon Islands	OT	Other
SLE	Sierra Leone	OT	Other
SLV	El Salvador	OT	Other
SMR	San Marino	EU	Europe
SOM	Somalia	OT	Other

SPM	Saint Pierre and Miquelon	NA	North America
SRB	Serbia	EU	Europe
STP	Sao Tome and Principe	OT	Other
SUR	Suriname	OT	Other
SVK	Slovakia	EU	Europe
SVN	Slovenia	EU	Europe
SWE	Sweden	EU	Europe
SWZ	Swaziland	OT	Other
SYC	Seychelles	OT	Other
SYR	Syrian Arab Republic	OT	Other
TCA	Turks and Caicos Islands	OT	Other
TCD	Chad	OT	Other
TGO	Togo	OT	Other
THA	Thailand	AS	Asia
TJK	Tajikistan	AS	Asia
TKL	Tokelau	OT	Other
TKM	Turkmenistan	AS	Asia
TLS	Timor-Leste	AS	Asia
TON	Tonga	OT	Other
TTO	Trinidad and Tobago	OT	Other
TUN	Tunisia	OT	Other
TUR	Turkey	EU	Europe
TUV	Tuvalu	OT	Other
TWN	Taiwan_Province of China	AS	Asia
TZA	Tanzania_United Republic of	OT	Other
UGA	Uganda	OT	Other
UKR	Ukraine	EU	Europe
UMI	United States Minor Outlying Islands	NA	North America
URY	Uruguay	OT	Other
USA	United States	NA	North America
UZB	Uzbekistan	AS	Asia
VAT	Holy See (Vatican City State)	EU	Europe
VCT	Saint Vincent and the Grenadines	OT	Other
VEN	Venezuela	OT	Other
VGB	Virgin Islands_British	OT	Other
VIR	Virgin Islands_USA	OT	Other
VNM	Viet Nam	AS	Asia
VUT	Vanuatu	OT	Other
WLF	Wallis and Futuna	OT	Other
WSM	Samoa	OT	Other
YEM	Yemen	OT	Other
ZAF	South Africa	OT	Other
ZMB	Zambia	OT	Other
ZWE	Zimbabwe	OT	Other

S5 Qualitative uncertainty estimates of global emissions

A qualitative indication of the emission variability at global level is reported in Table S6 and it is calculated as the relative difference between EDGARv6.1 and HTAP_v3 emissions by sector and pollutant. Further explanations are provided in section xx of the manuscript.

Table S6 – Variability of global emission estimates by sector and pollutant, calculated as the relative difference between HTAP_v3 emissions and the EDGARv6.1 estimates. Variability ranges are based on the qualitative classes defined in the EMEP/EEA Guidebook 2019 as low (L), low medium (LM), upper medium (UM), high (H).

Emission sector	Substance	(EDGARv6.1-HTAP_v3)/HTAP_v3, year 2000	(EDGARv6.1-HTAP_v3)/HTAP_v3, year 2018	varibility range, year 2000	varibility range, year 2018
HTAPv3_3_Energy	OC	69.3%	128.7%	UM	H
HTAPv3_3_Energy	BC	-1.9%	77.8%	L	UM
HTAPv3_3_Energy	SO2	-0.3%	44.5%	L	LM
HTAPv3_3_Energy	NOx	15.8%	24.4%	LM	LM
HTAPv3_3_Energy	CO	22.3%	20.7%	LM	LM
HTAPv3_3_Energy	NMVOC	34.9%	15.5%	LM	LM
HTAPv3_3_Energy	PM2.5	-16.4%	-1.2%	LM	L
HTAPv3_3_Energy	PM10	-17.2%	-2.7%	LM	L
HTAPv3_3_Energy	NH3	-1.9%	-39.5%	L	LM
HTAPv3_4.1_Industry	NMVOC	59.3%	96.4%	UM	UM
HTAPv3_4.1_Industry	SO2	-15.8%	85.5%	LM	UM
HTAPv3_4.1_Industry	OC	-24.0%	50.3%	LM	UM
HTAPv3_4.1_Industry	BC	-3.7%	47.8%	L	LM
HTAPv3_4.1_Industry	PM2.5	-46.6%	40.2%	LM	LM
HTAPv3_4.1_Industry	NOx	-1.6%	21.5%	L	LM
HTAPv3_4.1_Industry	PM10	-60.3%	-0.5%	UM	L
HTAPv3_4.1_Industry	CO	-25.8%	-2.6%	LM	L
HTAPv3_4.1_Industry	NH3	-53.7%	-54.2%	UM	UM
HTAPv3_4.2_Fugitive	CO	53.5%	64.1%	UM	UM
HTAPv3_4.2_Fugitive	SO2	31.1%	52.7%	LM	UM

HTAPv3_4.2_Fugitive	BC	36.7%	50.2%	LM	UM
HTAPv3_4.2_Fugitive	NH3	30.2%	19.4%	LM	LM
HTAPv3_4.2_Fugitive	NMVOC	10.7%	13.4%	L	L
HTAPv3_4.2_Fugitive	NOx	29.9%	8.9%	LM	L
HTAPv3_4.2_Fugitive	PM10	-0.6%	0.9%	L	L
HTAPv3_4.2_Fugitive	PM2.5	-29.0%	-23.0%	LM	LM
HTAPv3_4.2_Fugitive	OC	-65.0%	-51.1%	UM	UM
HTAPv3_4.3_Solvents	NMVOC	2.2%	-25.2%	L	LM
HTAPv3_4.3_Solvents	PM2.5	-69.8%	-60.2%	UM	UM
HTAPv3_4.3_Solvents	PM10	-74.5%	-67.6%	UM	UM
HTAPv3_4.3_Solvents	NH3	-99.8%	-99.6%	UM	UM
HTAPv3_5.1_Road_Transport	NH3	52.3%	80.2%	UM	UM
HTAPv3_5.1_Road_Transport	NOx	-4.2%	-16.4%	L	LM
HTAPv3_5.1_Road_Transport	CO	-21.3%	-47.0%	LM	LM
HTAPv3_5.1_Road_Transport	OC	-36.2%	-51.1%	LM	UM
HTAPv3_5.1_Road_Transport	NMVOC	-11.0%	-58.1%	L	UM
HTAPv3_5.1_Road_Transport	BC	-48.3%	-60.5%	LM	UM
HTAPv3_5.1_Road_Transport	PM2.5	-63.2%	-74.5%	UM	UM
HTAPv3_5.1_Road_Transport	SO2	-53.1%	-81.2%	UM	UM
HTAPv3_5.1_Road_Transport	PM10	-90.3%	-93.8%	UM	UM
HTAPv3_5.2_Brake_and_Tyre_wear	BC	26.1%	19.1%	LM	LM
HTAPv3_5.2_Brake_and_Tyre_wear	OC	-33.5%	-25.6%	LM	LM
HTAPv3_5.2_Brake_and_Tyre_wear	PM2.5	-57.1%	-48.0%	UM	LM
HTAPv3_5.2_Brake_and_Tyre_wear	PM10	-84.9%	-80.0%	UM	UM
HTAPv3_5.3_Domestic_shipping	NMVOC	249.9%	191.3%	H	H

HTAPv3_5.3_Domestic_shipping	CO	221.2%	188.7%	H	H
HTAPv3_5.3_Domestic_shipping	SO2	-5.5%	13.7%	L	L
HTAPv3_5.3_Domestic_shipping	PM2.5	11.4%	13.6%	L	L
HTAPv3_5.3_Domestic_shipping	PM10	11.1%	13.5%	L	L
HTAPv3_5.3_Domestic_shipping	BC	5.2%	11.3%	L	L
HTAPv3_5.3_Domestic_shipping	OC	6.3%	6.0%	L	L
HTAPv3_5.3_Domestic_shipping	NOx	-5.2%	3.3%	L	L
HTAPv3_5.3_Domestic_shipping	NH3	-41.5%	-20.9%	LM	LM
HTAPv3_5.4_Other_ground_transport	PM2.5	-34.5%	8.9%	LM	L
HTAPv3_5.4_Other_ground_transport	NH3	-13.8%	-17.4%	L	LM
HTAPv3_5.4_Other_ground_transport	NOx	-55.5%	-33.1%	UM	LM
HTAPv3_5.4_Other_ground_transport	PM10	-47.7%	-37.7%	LM	LM
HTAPv3_5.4_Other_ground_transport	OC	-71.8%	-41.7%	UM	LM
HTAPv3_5.4_Other_ground_transport	NMVOC	-80.8%	-64.6%	UM	UM
HTAPv3_5.4_Other_ground_transport	BC	-86.0%	-73.3%	UM	UM
HTAPv3_5.4_Other_ground_transport	CO	-82.6%	-82.3%	UM	UM
HTAPv3_5.4_Other_ground_transport	SO2	-83.8%	-84.0%	UM	UM
HTAPv3_6_Residential	PM10	30.2%	18.2%	LM	LM
HTAPv3_6_Residential	NH3	15.0%	4.9%	LM	L
HTAPv3_6_Residential	SO2	-8.0%	3.9%	L	L
HTAPv3_6_Residential	PM2.5	-7.4%	-9.5%	L	L

HTAPv3_6_Residential	NMVOC	-17.0%	-18.3%	LM	LM
HTAPv3_6_Residential	OC	-16.5%	-20.5%	LM	LM
HTAPv3_6_Residential	CO	-20.6%	-20.5%	LM	LM
HTAPv3_6_Residential	NOx	-39.0%	-28.8%	LM	LM
HTAPv3_6_Residential	BC	-41.6%	-40.3%	LM	LM
HTAPv3_7_Waste	NMVOC	78.1%	54.9%	UM	UM
HTAPv3_7_Waste	SO2	9.2%	7.4%	L	L
HTAPv3_7_Waste	NH3	-34.5%	-13.3%	LM	L
HTAPv3_7_Waste	PM10	-60.8%	-48.6%	UM	LM
HTAPv3_7_Waste	NOx	-50.5%	-57.3%	UM	UM
HTAPv3_7_Waste	PM2.5	-70.5%	-58.4%	UM	UM
HTAPv3_7_Waste	BC	-81.2%	-74.0%	UM	UM
HTAPv3_7_Waste	OC	-89.9%	-82.7%	UM	UM
HTAPv3_7_Waste	CO	-95.7%	-95.8%	UM	UM
HTAPv3_8.1_Agricultural_waste_burning	OC	7.5%	6.7%	L	L
HTAPv3_8.1_Agricultural_waste_burning	PM2.5	6.6%	6.1%	L	L
HTAPv3_8.1_Agricultural_waste_burning	CO	7.0%	5.8%	L	L
HTAPv3_8.1_Agricultural_waste_burning	PM10	5.6%	5.4%	L	L
HTAPv3_8.1_Agricultural_waste_burning	SO2	5.6%	5.1%	L	L
HTAPv3_8.1_Agricultural_waste_burning	NOx	5.4%	4.9%	L	L
HTAPv3_8.1_Agricultural_waste_burning	BC	3.8%	4.0%	L	L
HTAPv3_8.1_Agricultural_waste_burning	NH3	1.0%	2.7%	L	L
HTAPv3_8.1_Agricultural_waste_burning	NMVOC	-1.1%	0.3%	L	L

HTAPv3_8.2_Agriculture_livestock	NOx	11.5%	10.7%	L	L
HTAPv3_8.2_Agriculture_livestock	NMVOC	-14.7%	-9.4%	L	L
HTAPv3_8.2_Agriculture_livestock	NH3	-25.2%	-20.9%	LM	LM
HTAPv3_8.2_Agriculture_livestock	PM10	-33.8%	-26.7%	LM	LM
HTAPv3_8.2_Agriculture_livestock	PM2.5	-34.8%	-27.8%	LM	LM
HTAPv3_8.3_Agriculture_crops	NOx	13.1%	11.7%	L	L
HTAPv3_8.3_Agriculture_crops	NH3	16.6%	8.7%	LM	L
HTAPv3_8.3_Agriculture_crops	NMVOC	6.9%	6.8%	L	L
HTAPv3_8.3_Agriculture_crops	PM2.5	-82.1%	-77.8%	UM	UM
HTAPv3_8.3_Agriculture_crops	PM10	-92.6%	-91.6%	UM	UM

References

Klimont, Z., Kupiainen, K., Heyes, C., Purohit, P., Cofala, J., Rafaj, P., Borken-Kleefeld, J., and Schöpp, W.: Global anthropogenic emissions of particulate matter including black carbon, Atmos. Chem. Phys., 17, 8681-8723, 10.5194/acp-17-8681-2017, 2017.

Kurokawa, J. and Ohara, T.: Long-term historical trends in air pollutant emissions in Asia: Regional Emission inventory in ASia (REAS) version 3, Atmospheric Chemistry and Physics, 20, 12761-12793, https://doi.org/10.5194/acp-20-12761-2020, 2020.

O'Rourke, P. R., Smith, S. J., Mott, A., Ahsan, H., McDuffie, E. E., Crippa, M., Klimont, S., McDonald, B., Z., Wang, Nicholson, M. B, Feng, L., and Hoesly, R. M.: Community Emissions Data System (Version 04-21-2021), Zenodo. https://doi.org/10.5281/zenodo.4737769 2021.

Oreggioni, G. D., Mahiques, O., Monforti-Ferrario, F., Schaaf, E., Muntean, M., Guizzardi, D., Vignati, E., and Crippa, M.: The impacts of technological changes and regulatory frameworks on global air pollutant emissions from the energy industry and road transport, Energy Policy, 168, 113021, https://doi.org/10.1016/j.enpol.2022.113021, 2022.
